# Distinct response patterns of plants and soil microorganisms to agronomic practices and seasonal variation in a floodplain ecosystem

**DOI:** 10.3389/fmicb.2023.1094750

**Published:** 2023-01-26

**Authors:** Yanyan Yu, Hao Liu, Lanlan Zhang, Zhongjie Sun, Binghai Lei, Yuan Miao, Haiyan Chu, Shijie Han, Yu Shi, Junqiang Zheng

**Affiliations:** ^1^International Joint Research Laboratory for Global Change Ecology, School of Life Sciences, Henan University, Kaifeng, Henan, China; ^2^School of Science and Technology, Xinyang College, Xinyang, Henan, China; ^3^Yellow River Floodplain Ecosystems Research Station, Henan University, Kaifeng, Henan, China; ^4^State Key Laboratory of Soil and Sustainable Agriculture, Institute of Soil Science, Chinese Academy of Sciences, Nanjing, China; ^5^College of Resources and Environment, University of Chinese Academy of Sciences, Beijing, China

**Keywords:** floodplain, nitrogen and glyphosate, seasonal variation, plant traits, soil microorganisms

## Abstract

**Introduction:**

Climate change and anthropogenic activities are the greatest threats to floodplain ecosystems. A growing body of literature shows that floodplain ecosystems have experienced increased chemical fertilizer and pesticide loads, which will disturb the above and belowground ecosystems. However, we lack knowledge regarding the effects of such human activities on the vegetation and soil microbiomes in these ecosystems.

**Methods:**

In the present study, plant functional traits and Illumina Mi-Seq sequencing were to assess the impact of nitrogen fertilizer and glyphosate addition on the structure and function of the vegetation and soil microbiomes (bacteria, fungi, and protists) in a floodplain ecosystem, and to assess the influence of seasonal variation.

**Results:**

We identified distinct response mechanisms of plant and microbial communities to the addition of nitrogen fertilizer and glyphosate, and seasonal variation. Nitrogen fertilizer and glyphosate significantly affected plant diversity, aboveground and underground biomass, and C and N content and significantly changed the leaf area and plant stature of dominant plants. However, the addition of nitrogen fertilizer and glyphosate did not significantly affect the diversity and structure of bacterial, fungal, and protist communities. The application of nitrogen fertilizer could improve the negative effects of glyphosate on the functional traits of plant communities. The seasonal variation of floodplain has significantly changed the soil’s physical, chemical, and biological properties. Our results showed that compared with that in summer, the soil ecosystem multifunctionality of the floodplain ecosystem in autumn was significantly lower. Seasonal variation had a significant effect on plant diversity and functional traits. Moreover, seasonal variation significantly affected the community compositions, diversity, and structure of bacteria, fungi, and protists. Seasonal variation had a stronger impact on fungal community assembly than on that of bacteria and protists. In summer, the assembly of the fungal community was dominated by a deterministic process, while in autumn, it is dominated by a stochastic process. In addition, the negative association among bacteria, fungi, and protists has been strengthened in autumn and formed a more robust network to cope with external changes.

**Discussion:**

These results extended our understanding of the ecological patterns of soil microbiomes in floodplain ecosystems and provided support for enhancing the ecological barrier function and the service potential of floodplain ecosystems.

## 1. Introduction

Complex and natural environments have been altered to become simpler with (presumably) fewer stable ecosystems by the intensification of agriculture and the domestication of less productive land (Iglesias et al., 2021). This phenomenon involves the intensive use of chemical fertilizers and pesticides for yield improvement and weed chemical control. As a result of these changes, biodiversity and ecosystem stability have decreased (Viglizzo et al., 2011). The potential deleterious effects of the extensive use of chemical fertilizers and herbicide on ecosystems is a serious concern (Pizarro et al., 2016).

In agricultural practice, crop production is commonly increased using nitrogen fertilization; however, it also reduces the diversity of aboveground plant communities, leading to soil acidification, and the destruction of ecological sustainability (Ruan et al., 2016; Roley et al., 2018). Studies have shown that soil microorganisms are often sensitive to N fertilization (Ouyang and Norton, 2020; Sun et al., 2020, 2021). The increase in soil N availability can directly affect the microbial community by inhibiting the activities of enzymes and changing the community composition (Fierer et al., 2012; Yan et al., 2017; Li et al., 2021). Moreover, the addition of N fertilizer can indirectly affect the structure and diversity of microbial communities by changing plant communities and the physical and chemical properties of the soil (Zhao et al., 2015; Zeng et al., 2016; Hu et al., 2017), resulting in changes to riparian ecosystem function (Tian et al., 2015; Ling et al., 2016). As an example, an appropriate amount of N fertilizer can significantly increase plant growth and affect the biogeochemical process of the soil carbon cycle (Craig et al., 2021), leading to changes in the soil microbial community composition and structure by increasing the input of leaf and root litter to the soil (Lovelock et al., 2004; McKee et al., 2007; Dangremond et al., 2020). These changes in microbial community structure were accompanied by changes in the activity of enzymes involved in C, N, and P cycling (Craig et al., 2021; Sun et al., 2022b). The effect of N addition on soil microbial community structure is distinct in different ecosystems, which might be related to the amount of N input, the type of ecosystem, and local climate conditions (Chen et al., 2019). Until now, the responses of the soil microbiota to N in floodplain ecosystems have been largely unexplored.

In addition to nitrogen addition, the herbicide glyphosate [N-(phosphonomethyl)-glycine] is a non-selective organophosphate herbicide that is used worldwide in the production of many crops. However, the broad use of glyphosate can have adverse effects on non-target organisms, raising serious environmental concerns (Pizarro et al., 2016; López-Chávez et al., 2021). When it is sprayed or diffused onto the leaves and stems of the target plant, some will be absorbed (Galon et al., 2013), and the rest will end up in the soil through plant root physiology or leaching. After being absorbed by plants, glyphosate leads to plant metabolic disorders and death by blocking the shikimic acid biosynthesis pathway (Steinrücken and Amrhein, 1980). In fact, glyphosate blockage of the shikimic acid biosynthesis pathway of plants is also found in some bacteria and fungi (Helander et al., 2012). This negative effect on the shikimic acid biosynthesis pathway can cause a change in the soil microbial community structure, which might affect soil biological processes (Blagodatskaya and Kuzyakov, 2008). Certain taxa are sensitive to glyphosate, some are insensitive, and others might benefit from it (glyphosate metabolism releases phosphorus, carbon, or nitrogen); therefore, glyphosate can have variable and contradictory effects on soil microbial communities (Schlatter et al., 2017; Liu et al., 2018; Kepler et al., 2020).

The floodplain has important ecological and hydrological functions, such as forming corridors, buffer zones, and bank revetment (Meybeck, 1982), and plays a key role in biodiversity conservation, contamination filtration, soil erosion prevention, and flood control. However, floodplain ecosystems have experienced increased chemical fertilizer and pesticide loads resulting from anthropogenic activities. In addition, a floodplain characterized by irregular perturbations can have steep environmental gradients and is strongly influenced by the seasonal dynamics of the river (Fournier et al., 2020). Seasonal flooding is one of the unique seasonal factors of floodplain ecosystems, which is different from forest, grassland, farmland, and other ecosystems. The seasonal hydrological change of a floodplain is a crucial factor affecting plants and the microbial community (Rinklebe and Langer, 2010; Moche et al., 2015). These hydrological changes can affect plant community development and succession (Nam et al., 2015). Studies have shown that frequently changing redox conditions have a marked impact on the composition of microbial communities (Moche et al., 2015). The community assembly, structure, composition, and biomass of a microbial community are sensitive to the soil hydrological regime (Francioli et al., 2021). Thus, we might reasonably ask what is the impact of seasonal variation coupled with the addition of N fertilizer and glyphosate on plants and soil microorganisms?

To advance our knowledge on the effects of nitrogen fertilizer and glyphosate coupled with seasonal variation on vegetation and soil microbiomes in a floodplain ecosystem, the present study used DNA sequencing to investigate the diversity and structure of bacteria, fungi and protist communities. In addition, the functional characters of plants were determined. Increased soil N availability directly impacts the microbial community by altering the community composition and inhibiting enzyme activities (Li et al., 2021), while glyphosate acts on weeds mainly by interrupting the shikimate biosynthesis pathway in plants (Steinrücken and Amrhein, 1980). In addition, seasonal variations in temperature and moisture conditions could affect plant community structures (Yang et al., 2011), which in turn would influence the microbial community structure indirectly through shifts in the compositions of aboveground plant communities (Shi et al., 2020). In this study, we hypothesized that (1) Compared with plants, soil microorganisms would be more sensitive to nitrogen fertilizer and the effects of glyphosate on plant communities would more obvious than that on soil microorganisms; (2) Nitrogen fertilizer can partially neutralize the negative effect of glyphosate on plant communities; and (3) Seasonal variation would significantly affect both above- and below-ground ecosystem processes, and partially eliminate the effects of nitrogen fertilizer and glyphosate.

## 2. Materials and methods

### 2.1. Design of the experiments and collection of samples

**FIGURE 1:**
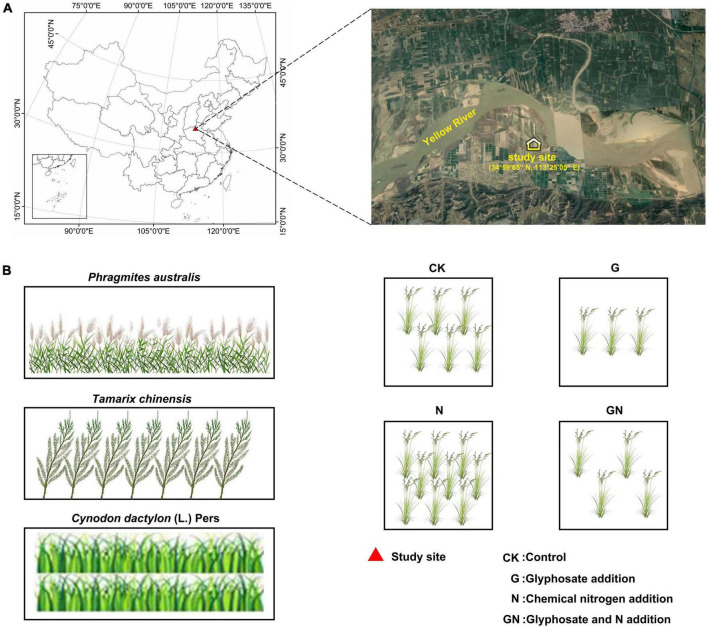
Maps of the sampling sites and a diagram of experimental design of this study. **(A)** The geographical distribution of the sampling sites; **(B)** the experimental design of this study.

We conducted the study at the Yellow River Floodplain Ecosystems Research Station (34°59′65″ N, 113°25′05″ E), Henan, China in 2019 (Figure 1). The study site is located in the Yellow River basin. The Yellow River has the largest sediment load and the highest sediment concentration in the world. The floodplain in the middle and lower reaches are characterized by unstable “wandering” distribution. The soil is a newly formed primary soil. The soil is salinized and alkalized, the sand content is high, and the porosity between particles is relatively large. At the same time, the soil’s water and fertilizer retention performance is weak, the soil was seriously nitrogen deficient. We chose three vegetation covers on the floodplain ecosystem: *Phragmites australis*, *Tamarix chinensis*, and *Cynodon dactylon* (L.) Pers. Each vegetation cover received four treatments: (i) Control (CK): plots with no N fertilizer and glyphosate added; (ii) Glyphosate addition (G): plots with glyphosate (the glyphosate dose was the equivalent of the application of 3 L ha^–1^ according to the recommended doses in the region); (iii) Chemical nitrogen addition (N): plots with chemical fertilizers comprising NH_4_NO_3_ (100 kg N ha^–1^year^–1^); and (iv) Glyphosate and N addition (GN): plots with combined application of N fertilizer and glyphosate. N fertilizer and glyphosate were added from November 2019 to May 2020. N fertilizer was added five times and glyphosate was added in twice. Doses of nitrogen fertilizer and glyphosate refer to the previous survey on commonly used doses of chemical fertilizers and herbicides in this area. Glyphosate and N were diluted using distilled water before application. An equivalent amount of distilled water was applied to the controls.

Four replicates were set for each treatment, with a total of 48 plots, each of which was 4 m × 4 m. Soil samples of 0–10 cm were collected in the summer (June) and autumn (November) of 2020. The study area experienced intermittent seasonal flooding from June to November every year, and the flooding frequency was four floods per year. The soil core device was cleaned before each sampling to avoid cross-contamination between samples. A total of 96 soil samples were collected (4 replications × 4 treatments × 3 vegetation communities × 2 seasons). The collected soil samples were divided into three parts. The first part was stored at −80°C for subsequent determination of the soil microbial community structure. The second part was stored at −4°C for later determination of the contents of glyphosate, nitrate (NO_3_^–^-N), and ammonium (NH_4_^+^-N). The third part was air-dried at room temperature, passed through a 0.25 mm mesh sieve, and subjected to chemical analysis.

### 2.2. Physicochemical analysis of the soil samples

A Vario Max CNS elemental analyzer (Elementar Analysensysteme GmbH, Hanau, Germany) was used to determine the total carbon (TC) and nitrogen (TN) contents in the soil. NO_3_^–^ and NH_4_^+^ were determined using a Discrete Auto Analyzer (SmartChem 200, WestCo Scientific Instruments Inc., Rome, Italy). Soil pH was measured using a pH meter (Sartorius PT-21, Shanghai, China) with 1:2.5 soil-to-ddH_2_O ratios. Fresh soil (15 g) was dried at 105°C for 48 h to measure soil moisture as drying-associated weight loss. Available potassium (AK), available calcium (ACa), available magnesium (AMg), available sodium (ANa), available copper (ACu), available iron (AFe), available manganese (AMn), and available zinc (AZn) were determined using an inductively coupled plasma emission spectrometer (ICP-MS) (Agilent, Santa Clara, CA, USA; 5100 ICP-OES). The Mo-Sb colorimetry method was used to determine the available phosphorus (AP) after the soil samples were extracted using NaHCO_3_ (0.5 M) (Bao, 2000). High performance liquid chromatography (HPLC) (e2695; Waters, Milford, MA, USA) was used to determine the glyphosate content.

### 2.3. Soil extracellular enzyme activities

In this study, the activities of six soil enzymes, including α-glucosidase (AG), β-glucosidase (BG), β-Xylosidase (BX), N-acetyl-β-glucosaminidase (NAG), alkaline phosphatase (ALP), and β-D-cellobioside (BCE), were determined using a fluorescence method (Saiya-Cork et al., 2002) with 96-well microplates (PerkinElmer Enspire, Waltham, MA, USA). The enzyme activity was calculated with reference to German et al. (2011).

### 2.4. Soil respiration and temperature

To measure soil respiration (R_*S*_), an 11 cm-diameter, 5 cm high polyvinyl chloride (PVC) tube was inserted into the soil at 2 cm in the center of each plot. The living plants in the tubes were removed at 1 day prior to measurement to eliminate aboveground plant respiration. Soil heterotrophic respiration (R_*H*_) was determined using an 11 cm-diameter, 60 cm high PVC tube. These PVC tubes cut off old plant roots and prevented the growth of new roots inside the tubes. The tubes were installed 1 month before the measurement, and all tubes were left at the site for the whole study period. There were no living plants or litter left in each tube during both respiration measurements. A LI-COR infrared gas analyzer (LI-8100, LI-COR Inc., Lincoln, NE, USA) was used to measure R_*S*_ and R_*H*_. Each soil respiration measurement took at total of 120 s, including a deadband of 30 s and an effective measurement time of 90 s. We monitored the soil temperature adjacent to each PVC tube at 0–10 cm deep using a thermocouple probe attached to the LI-8100 system.

### 2.5. Plant functional traits

We measured eleven plant functional traits: aboveground biomass, aboveground carbon and nitrogen content (C, N), underground biomass, root C and N content, plant stature (S), leaf area (LA), specific leaf area (SLA), leaf dry weight (LDW), and leaf dry matter content (LDMC). These measurements were carried out within a quadrat of 0.5 m × 0.5 m. We sorted all the above-ground living tissues in the quadrat by species. Plants were then dried for 24 h at 70°C and weighed as the aboveground biomass. The 0–10 cm roots of all species in the quadrat were collected, dried for 24 h at 70°C, and weighed as the underground biomass. After drying and weighing, we ground the aboveground and underground biomasses and filtered them through a 0.25 mm filter to determine the total N concentration, using a Vario MACRO Cube elemental analyzer (Elementar, Hanau, Germany). The height of each plant species was determined as the mean height of five randomly selected individuals. All individuals used for observation had their species typed using more than five individuals. Five healthy leaves for each species were selected in each quadrat, taken to the laboratory, immersed in water, and kept away from light for 12 h at 4°C. The excess water on the leaf surfaces was removed by suction and the leaves were weighed as the fresh weight of the leaves. Then, the leaf was scanned using a CanonLiDE 220 (Canon, Tokyo, Japan) and the leaf area (LA) was calculated using Image J1.48v (NIH, Bethesda, MD, USA). Drying and weighing of leaves were used to assess the LDW. The LDMC was calculated as LDW per unit fresh weight of the leaf. SLA was calculated as leaf area per unit dry mass.

### 2.6. Illumina MiSeq sequencing

Soil total DNA was extracted using an Omega Soil DNA Kit (M5635-02, Omega Bio-Tek, Norcross, GA, USA), following the manufacturer’s instructions. The V3–V4 hypervariable region sequences of bacteria were amplified by the primers 338F (ACTCCTACGGGAGGCAGCAG) and 806R (GGACTACHVGGGTWTCTAAT). The Internal Transcribed Spacer 1 (ITS1) of fungi was amplified by the primers ITS5F (GGAAGTAAAAGTCGTAACAAGG) and ITS1R (GCTGCGTTCTTCATCGATGC). The 18S V4 hypervariable region sequences of soil protists were amplified by the primers 547F (CCAGCASCYGCGGTAATTCC) and V4R (ACTTTCGTTCTTGATYRA). Protists mainly include Stramenopiles, Amoebozoa, Alveolata, Archaeplastida, Rhizaria, Excavata and Opisthokonta (We removed *Rhodophyta*, *Streptophyta*, *Fungi*, *Opisthokonta_X*, *Metazoa*, and ambiguous taxa in Eukaryotes from the 18S rDNA amplicon sequence). The MiSeq system carried out pair-end sequencing with the Illumina MiSeq Reagent Kit v3 in Shanghai Personal Biotechnology Co., Ltd. (Shanghai, China). All sequencing was accomplished using the Illumina MiSeq-PE300 platform (Illumina, San Diego, CA, USA). QIIME 2 was used to conduct microbiome bioinformatics (Bolyen et al., 2018). The raw reads with paired ends were demultiplexed using the demux plugin, and then the primers were cut using the cut adapt plugin (Martin, 2011). The sequences were quality-filtered and denoised using the DADA2 process in the settings (Callahan et al., 2016). We used the learnErrors, derepFastq, dada, and mergePairs methods with default settings to manage sequence quality and eliminate all sequencing errors. The singletons’ amplicon sequence variants (ASVs) were then eliminated. Following that, the taxonomic identities of bacteria, fungi, and protists were identified using the Silva v132 database^1^ (Quast et al., 2013), the UNITE database (Release 8.0)^2^ (Koljalg et al., 2013), and the PR2^3^ (Guillou et al., 2012), respectively.

The raw sequencing data were uploaded to the Sequence Read Archive (SRA) database at the National Center for Biotechnology Information, with the BioProject numbers: PRJNA876613, PRJNA877715, and PRJNA877765 for the bacteria, fungi, and protist sequences, respectively.

### 2.7. Assessing multifunctionality

We included 20 ecosystem functions that are regulated by soil organisms under a wide range of ecosystem services: AG, BG, BX, NAG, ALP, BCE, Soil TC, TN, AK, ACa, AMg, ANa, ACu, AFe, AMn, AZn, AP, pH, R_*S*_, and R_*H*_. The detailed description of ecosystem functions and their contributions to ecosystem services are provided in Supplementary Table 1. For the detailed description of ecological functions, refer to Maestre et al. (2022).

The values for ecosystem functions vary widely; therefore, all the variables were standardized to a common scale ranging from 0 to 1 as follows: STD = (X-Xmin)/(Xmax-Xmin); in which STD is the standardized variable and X, Xmin, and Xmax are the target variable, its minimum value, and its maximum value across all samples. To acquire a quantitative multifunctionality index for each sample, we used the average multifunctionality index (Jiao et al., 2022).

### 2.8. Statistical analysis

The soil biological, chemical, and physical properties were compared between nitrogen fertilizer, glyphosate addition, and seasonal variation using analysis of variance (ANOVA) and pairwise *t*-tests. These tests were implemented using SPSS 24 (IBM Corp., Armonk, NY, USA).

We calculated Faith’s phylogenetic diversity (PD), observed species, and Shannon index (Fungi) to evaluate how nitrogen and glyphosate treatment and seasonal variation affected microbial alpha-diversity. Non-metric multidimensional scaling (NMDS) was used to visualize dissimilarities in the community composition. The Mantel test was used to analyze the correlations between the soil microbial community and physicochemical properties. In addition, variance partitioning based on redundancy analysis (RDA) was carried out to assess the contribution made by soil properties to the structure of the microbial community. Based on the Bray-Curtis dissimilarity index, permutational multivariate analysis of variance (PERMANOVA) was carried out to evaluate the effects of nitrogen fertilizer, glyphosate addition, and seasonal variation on the microbial community structure, with each test using 999 permutations.

The underlying mechanism(s) that affected the community assembly were computed using the mean nearest taxon distance (MNTD) and the mean pairwise phylogenetic distance (MPD) (Stegen et al., 2012). Detailed information is provided in the Supplementary material.

We constructed co-occurrence networks in June and November, respectively, comprising soil phylotypes from bacteria, fungus, and protists. To decrease the effect on the network structure of site-specific amplicon sequence variants (ASVs), core microbial ASVs were selected that were shared by >60% of the samples, with a mean of relative abundance >0.1%, after which SparCC was used to generate the co-occurrence networks. To construct the network, nodes were selected that had an absolute value of correlation greater than 0.6 and a *p*-value less than 0.01. The network was visualized using Cytoscape 3.8.2 (Shannon et al., 2003). The topology of the networks was characterized using a set of determined metrics, including the betweenness of centrality and modularity, the proximity of centrality, the degree of centrality, and the number of vertices and edges. The Z-score and C-score were used to identify the core species. We divided the microbial community into four categories: network hubs (Z-score > 2.5, C-score > 0.62), module hubs (Z-score > 2.5, C-score < 0.62), connectors (Z-score < 2.5, C-score > 0.62), and peripherals (Z-score < 2.5, C-score < 0.62) (Olesen et al., 2006). Change in the natural network connectivity were assessed by removing nodes in the network randomly and equally, followed by evaluation of the network robustness according to natural connectivity.

Statistical analyses were carried out using the R environment (v3.5.1),^4^ unless otherwise stated.

## 3. Results

### 3.1. Effects of agronomic practices and seasonal variation on soil physical, chemical, and biological properties

N fertilizer significantly increased the ANa (p < 0.05); however, N fertilizer and glyphosate had no significant effect on other measured soil properties in summer (Supplementary Table 2). Except for TC, AP, pH, and NO_3_, agricultural management did not result in significant differences in the measured soil variables in autumn. We found a 50% decrease in the glyphosate content under N fertilizer addition compared with that in the control. In summer, agricultural management did not affect the activities of enzymes, except that N fertilizer resulted in a significant increase in the activity of AP (Supplementary Figure 1). In autumn, a significant decrease in BG under N addition was observed. In summer, glyphosate addition significantly reduced R_*S*_, while N addition significantly increased R_*S*_ (Supplementary Figure 2). The R_*S*_ of the GN treatment group was between that of the glyphosate and N fertilizer alone groups. None of the agricultural managements had a significant effect on R_*H*_. N fertilizer and glyphosate did not significantly affect soil ecosystem multifunctionality (Supplementary Figure 3).

**FIGURE 2:**
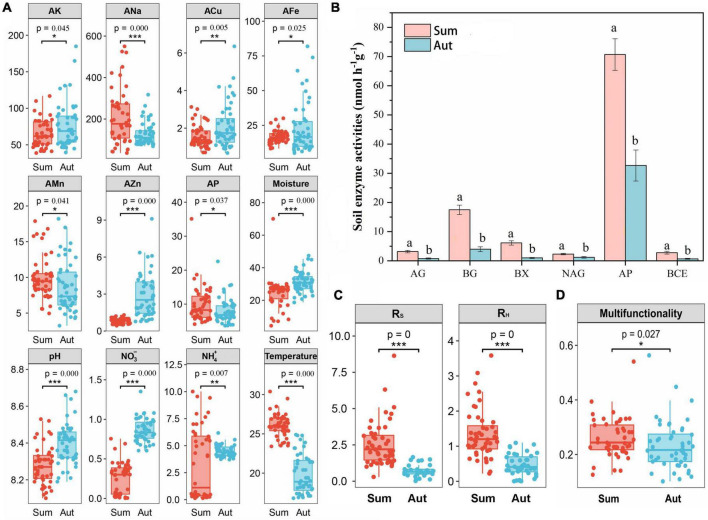
Soil physical, chemical, biological properties and multifunctionality. **(A)** Soil properties in summer and autumn; **(B)** soil extracellular enzyme activities in summer and autumn; **(C)** soil respiration in summer and autumn. **(D)** Soil multifunctionality in summer and autumn. Sum, summer; Aut, autumn; R_S_, soil respiration; R_H_, soil heterotrophic respiration; AK, available potassium; ACa, available calcium; AMg, available magnesium; ANa, available sodium; ACu, available copper; AFe, available iron; AMn, available manganese; AZn, available zinc; AP, available phosphorus. AG, α-glucosidase; BG, β-glucosidase; BX, β-Xylosidase; NAG, N-acetyl-β-glucosaminidase; ALP, alkaline phosphatase; BCE, β-D-cellobioside. Data (a and b) that do not share a letter are significantly different between treatments (*P* < 0.05). *P < 0.05, **P < 0.01, and ***P < 0.001.

Seasonal variation significantly influenced the soil physicochemical properties (Figure 2A and Supplementary Table 3). In general, AK, ACu, AFe, AZn, soil moisture, pH, NO_3_, and NH_4_ increased significantly in autumn (*p* < 0.05). We also noticed that the content of glyphosate in all soil samples decreased in autumn compared with that in summer. Generally, in autumn, the activities of all six enzymes were significantly reduced compared with those in summer (Figure 2B). There were significant reductions in R_*S*_ and R_*H*_ in autumn compared with those in summer (Figure 2C). Significantly reduced soil ecosystem multifunctionality was observed in autumn compared with that in summer (Figure 2D).

### 3.2. Responses of plant species diversity and functional traits to agronomic practices and seasonal variation

The application of glyphosate eliminated most weeds in the floodplain ecosystem, such as *Aster tataricus* L. f, *Sonchus wightianus* DC, *Setaria viridis* (L.) Beauv, *Cyperus serotinus* Rottb, and *Erigeron canadensis* L. Whereas, *Tamarix chinensis* Lour, *Phragmites australis*, and *Cynodon dactylon* (L.) Pers have developed tolerance against this herbicide (Supplementary Figure 4).

In summer, glyphosate significantly reduced plant diversity, while N fertilizer significantly increased plant diversity. The plant diversity under GN treatment was between that of glyphosate and N fertilizer treatment alone (Supplementary Figure 5). In autumn, glyphosate and N fertilizer had no significant effect on plant diversity.

**FIGURE 3:**
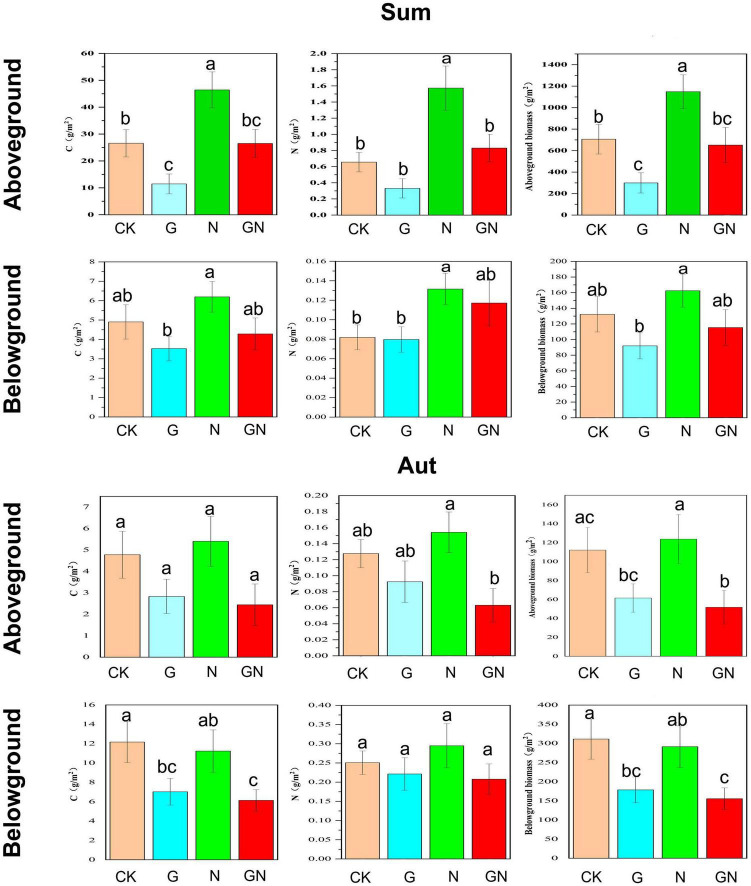
Plant functional traits with each treatment group (C content, N content and biomass of aboveground and belowground). Sum, summer; Aut, autumn; CK, control; G, glyphosate addition; N, chemical nitrogen addition; GN, glyphosate and N addition. Data (a, b, ab, and bc) that do not share a letter are significantly different between treatments (*P* < 0.05).

In summer, plant aboveground biomass and the aboveground C and N content showed significantly increasing trends under N fertilizer treatment, but significantly decreasing trends under glyphosate treatment (Figure 3). Aboveground biomass and the aboveground C and N content values under GN treatment were between those of the N fertilizer and glyphosate alone groups. Meanwhile, N fertilizer significantly increased the root N content, while the addition of glyphosate significantly reduced the underground biomass and root carbon content. The underground biomass and root C and N content values under GN treatment were between those of the groups treated with N fertilizer and glyphosate alone. N fertilizer significantly increased the LA of *Phragmites australis*, and significantly increased the S of *Phragmites australis* and *Cynodon dactylon* (L.) Pers (Supplementary Figure 6). Glyphosate significantly reduced the LA and S of *Phragmites australis*. The LA value of *Phragmites australis* and the S values of *Phragmites australis* and *Cynodon dactylon* (L.) Pers under GN treatment were between those of the groups treated with N fertilizer and glyphosate alone.

In autumn, N fertilizer increased the plant aboveground biomass and aboveground C and N content, while glyphosate treatment reduced the above traits (Figure 3). Meanwhile, the addition of glyphosate significantly reduced the underground biomass and root C content. The experimental treatments did not significantly alter the LDMC, LA, SLA, and S of *Cynodon dactylon* (L.) Pers (Supplementary Figure 6).

**FIGURE 4:**
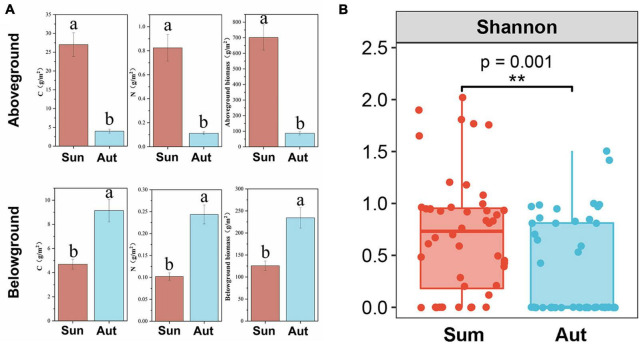
Plant functional traits and diversity in summer and autumn. **(A)** C content, N content and biomass of aboveground and belowground in summer and autumn; **(B)** plant species diversity in summer and autumn. Sum, summer; Aut, autumn; CK, control; G, glyphosate addition; N, chemical nitrogen addition; GN, glyphosate and N addition. Data (a and b) that do not share a letter are significantly different between treatments (*P* < 0.05). **P < 0.01.

Seasonal variation had a significant effect on plant growth and plant diversity (Figure 4). A significant reduction in plant diversity, aboveground biomass, and aboveground C and N content was found in autumn; however, seasonal variation caused an increase in underground biomass, and root C and N content.

### 3.3. Impacts of agronomic practices and seasonal variation on microbial communities

N and glyphosate addition did not significantly affect the abundance and diversity of bacterial, fungal, and protist communities in summer and autumn (Supplementary Figure 7). Compared with that in summer, the bacterial community showed a significant decrease in Faith’s PD index in autumn, whereas Faith’s PD index of the protist community increased significantly (Supplementary Figure 7).

Furthermore, large differences in bacterial, fungal, and protist community compositions at the phylum and genus level were observed between summer and autumn (Supplementary Figure 8). Linear discriminant analysis (LDA) effect size (LEfSe) was used to identify dominant phylotypes of bacteria, fungi, and protist, as indicative biomarkers of seasonal variation (Supplementary Figure 9). In summer, higher abundances of members affiliated with the phyla Actinobacteria, Bacteroidetes, Patescibacteria, Acremonium, Ascomycota, Glomeromycota, Olpidiomycota, Sarocladium, Dinoflagellata, Haptophyta, Radiolaria, Opalozoa, and Stramenopiles_X were observed (*p* < 0.05) compared with those in autumn. By contrast, members of the phyla Chloroflexi, Cyanobacteria, Nitrospirae, Cadophora, Rozellomycota, and Chytridiomycota were significantly more abundant in autumn (*p* < 0.05).

There was no significant difference in bacterial, fungal and protist community structure between different treatments; however, seasonal variation significantly affected the community structure of bacteria, fungi, and protists (Supplementary Figure 10 and Supplementary Table 4). Mantel analysis showed that TN, TC, AK, ACa, AMg, ANa, ACu, AFe, AMn, and AZn correlated significantly with the bacterial community structure. AK, AMg, ACu, AFe, AMn, and AZn correlated significantly with the fungal community structure. TC, AK, AMg, ANa, ACu, AFe, AMn, AZn, moisture, pH, and nitrate correlated significantly with the protist community structure (Supplementary Table 5). The RDA of the bacteria, fungi, and protist community structures at the general level showed that the communities were divided into two groups according to the seasonal variation (Supplementary Figure 11). Soil properties were important factors affecting the microbial communities in the autumn.

### 3.4. Influence of seasonal variation on the inter-kingdom association network patterns and assembly of the microbial community

**FIGURE 5:**
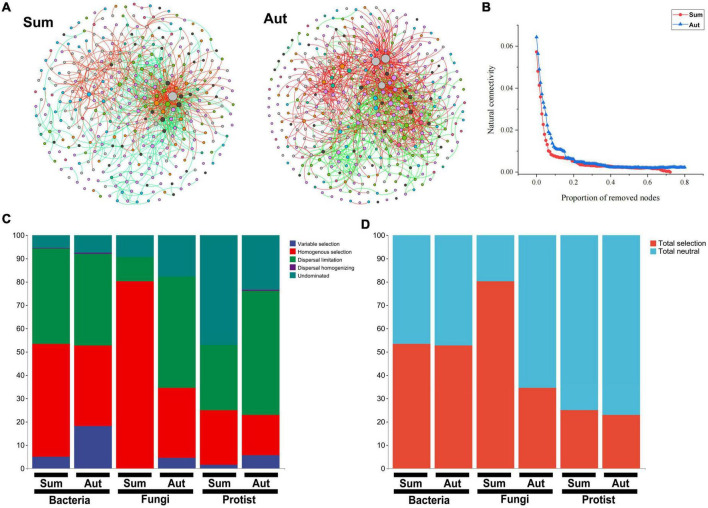
The inter-kingdom association network patterns and assembly of the microbial community in summer and autumn. **(A)** A network of soil microbial amplicon sequence variants (ASVs) found in summer and autumn based on correlations. Each node represents an ASV, and the edges connecting nodes represent either positive (red) or negative (black) correlations inferred using the SparCC approach using ASVs abundance profiles (pseudo *p* < 0.05, correlation values < – 0.6 or >0.6). **(B)** Network stability in summer and autumn. **(C)** The relative contribution (%) of five ecological processes. **(D)** The relative contribution (%) of determinism and stochasticity on the microbiome assembly based on the β-Nearest Taxon Index (βNTI) values. Sum, summer; Aut, autumn.

There were more nodes and edges of the microbial network in autumn than in summer, and average number of neighbors, network density, and the negative correlation ratio were also higher than those in summer (Figure 5A and Supplementary Table 6). ASVs belonging to Eurotiomycetes, Blastocatellia-(Subgroup-4), Latescibacteria, Alphaproteobacteria, KD4-96, Subgroup-6, MB-A2-108, and Gemmatimonadetes were identified as hubs or keystone species in the network in summer. By contrast, in autumn, ASVs belonging to Alphaproteobacteria, Gemmatimonadetes, Gammaproteobacteria, Thermoleophilia, Gammaproteobacteria, MB-A2-108, and KD4-96 formed the hubs or keystone species in the network (Supplementary Table 7). Notably, the autumnal network robustness was higher than that in summer (Figure 5B).

In autumn, total neutral processes contributed more to the assembly of bacterial, fungal and protist communities than in summer (Figures 5C, D and Supplementary Table 8). Among bacteria, fungi, and protists, the stochastic process made a higher contribution to the protist community than it did to the bacterial and fungal communities. Seasonal variation had a stronger effect on the assembly of fungal communities than that of bacterial and protist communities.

## 4. Discussion

### 4.1. The effects of nitrogen and glyphosate on plant communities were more obvious than that on soil microorganisms

Contrary to our first hypothesis, we found that glyphosate and N fertilizer applied at the recommended doses significantly changed the plant community, whereas, in general, they had no significant effects on the diversity and community structure of soil microorganisms. Consistent with previous studies, the changes in plant functional traits showed that N fertilizer significantly increased the plant aboveground biomass, aboveground carbon, nitrogen content, and root nitrogen content, and significantly enhanced the LA and S of dominant species. In contrast, glyphosate significantly reduced the aboveground biomass, carbon, nitrogen content, underground biomass, and root carbon content. However, in contrast to previous studies, we found that nitrogen fertilizer and glyphosate had no significant effect on the diversity and community structure of soil microorganisms. Similarly, we did not observe changes in the absolute activity of soil extracellular enzymes and soil heterotrophic respiration, which was possibly related to the lack of variation in soil physicochemical properties. In this study, N and glyphosate had no significant impact on the main soil properties.

Previous studies showed that glyphosate application induces a variety of soil biota responses, including inhibitory and stimulatory effects (Nguyen et al., 2016; Kepler et al., 2020). Several factors might influence the effects of glyphosate on soil microbial diversity and activity, including different rates and formulations of applied herbicides, plant presence or absence, and variations in the pH and organic carbon contents of soils (Iglesias et al., 2021). Compared with other studies, the reasons for the lack of effects documented here are as follows: The Yellow River has the largest sediment load and the highest sediment concentration in the world. The floodplain in the middle and lower reaches are characterized by unstable “wandering” distribution. Therefore, the soil is a newly formed primary soil, and the soil in the study area was seriously nitrogen deficient (Chen et al., 2022). The soil is salinized and alkalized, and the sand content is high. At the same time, because of the lack of organic colloids, the soil’s water and fertilizer retention performance is weak, causing serious loss of water and fertilizer (Chen et al., 2022). Nitrogen fertilizer and glyphosate are difficult to fix in soil. Although the exposure of N fertilizer and glyphosate may lead to the changes of soil microbial communities over time, the microbial groups showed high diversity and interconnectedness, which were likely to make their communities resistant or resilient to small disturbances (Weaver et al., 2007; Schlatter et al., 2017). Therefore, recovery of the communities to a pre-disturbance state is likely to be rapid, the impact of N fertilizer and glyphosate on microbial community functions is small. In addition, the low volumes and application rates of glyphosate and N fertilizer might also affect the response of soil microorganisms. A recent meta-analysis revealed that the impact of glyphosate on soil microorganisms is highly variable and depends upon specific experimental parameters, such as the dose of glyphosate applied (Nguyen et al., 2016). The different doses of glyphosate used in the present study might account for the observed lack of effects compared with previous studies. A study showed that treatment with glyphosate at 2.5 mg L^–1^ for 15 days had no significant effect on the chemical and physical state of the microbiota or the composition of its main species; however, a metatranscriptomic analysis revealed that enhanced expression of genes associated with catabolism, transport, secondary metabolites biosynthesis, and translation occurred in the microbial community, which were possibly related to their ability to withstand glyphosate contamination (Lu et al., 2020). Future experiments might wish to consider tracking the effects of nitrogen fertilizer and glyphosate on microbial communities over a longer period of time, and exploring the potential response of genes at the transcriptome level.

The different response modes of plants and soil microorganisms to N fertilizer and glyphosate also indicated that in the floodplain ecosystem, N fertilizer and glyphosate directly affected plants, and then may indirectly affected soil microbial community. Research shows that N fertilizer addition can indirectly affect the structure and diversity of microbial communities by changing the soil physical and chemical properties and plant communities (Zeng et al., 2016; Hu et al., 2017). Glyphosate is directly sprayed on the leaves and stems of target plants, and changes in plant characteristics will change the microbial community interacting with them (Weems et al., 2021). A study showed that, in the agricultural landscapes of the Pampas region in South America, the application of N fertilizer and glyphosate at recommended doses had slight stimulation on plant growth, but had no significant impact on the soil microbial functional composition and diversity (Iglesias et al., 2021). Our results showed that the changes of plant diversity and functional traits caused by the N fertilizer and glyphosate are asynchronous with the changes of soil microbial diversity and community structure.

### 4.2. Coupling effect of nitrogen fertilizer and glyphosate combined application on plant communities

Although glyphosate and N fertilizer do not significantly change the content of glyphosate in the soil, it should be noted that N fertilizer addition can reduce the glyphosate content in soil. The interception and absorption of glyphosate by plants, especially wild glyphosate-tolerant plants, can significantly promote the removal of glyphosate (López-Chávez et al., 2021). The promotion by N fertilizer of plant diversity and biomass will inevitably strengthen the efficiency of glyphosate removal. Our results suggested that in the floodplain ecosystem, N fertilizer could alleviate the environmental concern caused by the widespread use of glyphosate to a certain extent.

In addition to reducing the concentration of glyphosate in the soil, we also noticed a significant effect of N fertilizer in counteracting the effects of glyphosate on plant communities. Studies have shown that glyphosate can eliminate many weeds, such as Amaranthaceae, Asteraceae, Cucurbitaceae, Brassicaceae, Cyperaceae, Commelinaceae, Convolvulaceae, Euphorbiaceae, Fabaceae, Lamiaceae, Malvaceae, Poaceae, Portulacaceae, Rubiaceae, Sapindaceae, and Solanaceae (Gazziero, 2015). Therefore, in the floodplain ecosystem, the use of glyphosate will lead to a reduction in plant community diversity. A previous study showed that the diversity of aboveground plant communities was reduced by long-term inorganic N fertilizer application (Craig et al., 2021); however, the present study found that N fertilizer increased the diversity of aboveground plant communities, and the combined application of N fertilizer and glyphosate helped to alleviate the reduction of plant community diversity caused by glyphosate.

In addition, in terms of plant aboveground biomass, aboveground C and N content, underground biomass, and root C and N content, the combined application of N fertilizer and glyphosate effectively neutralized the negative impact of glyphosate on the above characteristics. As documented in other studies, herbicides applied to the field after plant emergence might reduce plant growth, thus counteracting the expected benefits to crops (Iglesias et al., 2021). Our study showed that considering the effect of treatment with nitrogen fertilizer and glyphosate together on plant communities, the combined application of N fertilizer and glyphosate could alleviate the inhibitory effect of glyphosate on plant traits.

### 4.3. Seasonal variation caused a joint change in plant and soil microbial communities

Seasonal variation significantly reduced plant diversity, the aboveground biomass, and the aboveground carbon and nitrogen content. The reason for this phenomenon is that the floodplain ecosystem experiences a seasonal flooding process every year. Plant performance is affected negatively by flooding, such as by reducing shoot growth, the number of leaves, and photosynthesis (Francioli et al., 2021). Flooding-induced anoxia markedly affects plant growth (Francioli et al., 2021). By contrast, the underground biomass and the root carbon and nitrogen content increased further after the growing season. Additionally, in ecosystems, there is high seasonal variation in plant growth resulting from significant seasonal differences (e.g., precipitation and temperature) (Shi et al., 2020). The trends in plant biomass followed a seasonal pattern of maximum in summer, a decrease in autumn, and a minimum after winter (Fiore-Donno et al., 2019).

Seasonal variation in soil and plant communities could directly or indirectly influence the belowground microbial communities (Shi et al., 2020). Our study further revealed that seasonal variation significantly affected the multifunctionality of the soil ecosystem and the community structures of bacterial, fungal and protist. In our study, the physicochemical conditions of the soil were altered strongly by seasonal variation. Importantly, soil nutrients in the floodplain vary according to the deposition of sediment carried by seasonal flooding (Chen et al., 2022). Waterlogging frequently causes changes in soil pH (Hemati and Jalali, 2017), which has been acknowledged as a major factor affecting microbiota structures across a wide range of ecosystems (Bardelli et al., 2017).

The quantity and community composition of soil microorganisms are influenced by soil physicochemical properties, thereby directly and indirectly affecting the activities of soil enzymes (Fan et al., 2021). Soil enzymatic activities are intimately related to soil microbial biomass and soil physicochemical properties (e.g., soil organic matter, soil pH, soil aggregate, and soil moisture and temperature) (Qi et al., 2016). In addition, the diverse types of vegetation caused by seasonal variation also directly affect soil microorganisms and enzymatic activities by influencing plant-soil metabolism (Fan et al., 2021).

Overall, these results confirmed our third hypothesis, emphasizing the fundamental effects of seasonal variation on soil and plant properties, which may be correlate strongly with soil microbial communities and ecological functions.

### 4.4. Seasonal variation caused shifts in microbial community assembly and co-occurrence networks

To determine the mechanism by which seasonal variation affects microbial communities, we further explored the co-occurrence networks and assembly of the microbial community. The present study showed that compared with that in summer, the scale of the network was larger in autumn, which also indicated that the complexity of the network was enhanced. In addition, we found that seasonal variation reshaped the microbial hierarchical interactions in the floodplain ecosystem. Compared with summer, microorganisms adjusted their partnership through trophic interactions, thereby strengthening the negative association between members in the network. The increase in the negative correlation ratio indicated an increase in the competitive relationship among soil microbial communities. Studies suggested that individuals that are linked positively in the network might show synchronous reactions to changes in the environment, producing positive feedback and co-oscillation (Coyte et al., 2015). Negatively linked individuals might help to modulate community fluctuations in response to disturbances, thereby enhancing network stability (Coyte et al., 2015). Natural connectivity-based robustness tests demonstrated that in autumn, the network was highly robust. This research demonstrated the positive response of microorganisms to seasonal interference. Studies have shown that seasonal variation has a significant impact on soil microbial communities through soil moisture, temperature, and the amount of litter (Chemidlin Prevost-Boure et al., 2011; Gruyter et al., 2020). In this study, seasonal flooding, as an important feature of seasonal variation in the floodplain, increased the difference in the co-occurrence networks of soil microbial communities.

In autumn, stochastic processes were mainly responsible for the assembly of soil microbial eukaryotic communities. Indeed, floods deposit propagules from outside the floodplain and move soil materials between habitats, thereby increasing dispersal and the stochastic potential. Compared with larger organisms (fungi and/or protist), smaller organisms (i.e., bacteria) are subject to less filtration by the environment, because smaller organisms have higher metabolic capacities and greater environmental tolerance (Wu et al., 2018). Some studies also proposed that not only the size of the body, but also the dispersal mode determines the metacommunity structure (De Bie et al., 2012; Sun et al., 2022a). For example, some fungi are spread more easily by the wind than bacteria (Dressaire et al., 2016). Protists have greater mobility in response to environmental interference (Yu et al., 2022), which might lead to greater dispersal abilities of fungi and protist compared with those of bacteria, especially on a small scale. The present study showed that after seasonal variation, such as intermittent seasonal flooding, fungi and protists were filtered less by the environment than bacteria. Moreover, the fungal community suffered from a more severe impact of seasonal variation than the bacterial and protist communities, which suggested that the assembly of the fungal community is more sensitive to seasonal variation. Previous studies revealed that in the microbiome, protists were the most sensitive to seasonal variation, because their communities changed most significantly between seasons in agroecosystems (Zhao et al., 2019). The seasonal variation of soil moisture caused by the seasonal flooding process of the floodplain might explain the seasonal variation of fungal community assembly, because the formation of fungal communities is driven mainly by moisture and nutrient availability (Peay et al., 2016). In autumn, the contributions (%) of homogenous selection to fungal and protist community assembly decreased, while the proportion of dispersal limitation increased. Homogenous selection will lead to more similar phylogenetic diversity of the community, while dispersal limitation will lead to more-dissimilar structures among communities (Zhou and Ning, 2017). The results of MDNS analysis also confirmed the above view, i.e., the heterogeneity of fungal and protist communities was higher in autumn.

## 5. Conclusion

Our findings indicated that the effects of nitrogen and glyphosate on plant communities would more obvious than that on soil microorganisms. The application of N fertilizer could alleviate the inhibitory effect of glyphosate on plant traits. The seasonal variation of floodplain has significantly changed the soil ecosystem multifunctionality. Seasonal variation also caused a joint change in plant and soil microbial communities. Seasonal variation significantly affected the diversity and functional traits of plant as well as the community compositions, diversity and structure of bacteria, fungi, and protists. Our study provided useful insights into the soil ecology of floodplain ecosystems, and highlighted the value of accurate estimations of the structure and complexity of the assembly processes of soil microbial communities under temporal dynamics within a complex ecosystem. Plant and soil microbial communities are important for functioning ecosystems, which is related to strengthening the ecological barrier function of floodplains and improving their service potential.

## Data availability statement

The datasets presented in this study can be found in online repositories. The names of the repository/repositories and accession number(s) can be found in the article/Supplementary material.

## Author contributions

JZ and SH conceived the presented idea and received important feedback from YY, HL, LZ, ZS, BL, YM, HC, and YS. ZS and BL carried out the field management. JZ, YY, HL, and LZ carried out the sampling collections, DNA preparation, and chemical measurement. JZ, HC, and YS carried out the MiSeq sequencing process and data analysis. YY and JZ wrote the manuscript with help from YS and YM. All authors discussed the methods and results and contributed to the final manuscript.

## Footnotes

1
http://www.arb-silva.de

2
https://unite.ut.ee/

3
https://figshare.com/articles/PR2-rRNA-gene-database/3803709

4
http://www.r-project.org/
