# Supplementary material for: Distinct response patterns of plants and soil microorganisms to agronomic practices and seasonal variation in a floodplain ecosystem
Source: Front Microbiol. 2023 Jan 26;14:1094750. doi: 10.3389/fmicb.2023.1094750 (PMC9909268; doi:10.3389/fmicb.2023.1094750)
Supplement: Supplementary file 1 [file Data_Sheet_1.docx]

**Supporting Information**

**Title:** Distinct response patterns of plants and soil microorganisms to agronomic practices and seasonal variation in a floodplain ecosystem

Yanyan Yu^1,2,3,6^, Hao Liu^1,3,6^, Lanlan Zhang^1,3^, Zhongjie Sun^1,3^, Binghai Lei^1,3^, Yuan Miao^1,3^, Haiyan Chu^4,5^, Shijie Han^1,3^, Yu Shi^1,3*^, Junqiang Zheng^1,3^^*^

^1^International Joint Research Laboratory for Global Change Ecology, School of Life Sciences, Henan University, Kaifeng, Henan, 475004, China.

^2^ School of Science and Technology, Xinyang College, Xinyang, Henan, 464000, China.

^3^ Yellow River Floodplain Ecosystems Research Station, Henan University, Xingyang, Henan, 450103, China.

^4^ State Key Laboratory of Soil and Sustainable Agriculture, Institute of Soil Science, Chinese Academy of Sciences, 71 East Beijing Road, Nanjing 210008, China.

^5^ University of Chinese Academy of Sciences, Beijing 100049, China.

^6^These authors contributed equally: Yanyan Yu and Hao Liu

******Corresponding authors*:** Yu Shi and Junqiang Zheng

**Tel:** 86-25-86881356

**E-mails:** Junqiang Zheng: zhjq79@yahoo.com; Yu Shi: [yshi@henu.edu.cn](mailto:yshi@henu.edu.cn)

**This file includes:**

**Fig S1 to S11**

**Table S1 to S8**

**Experimental procedure**

**Supporting Results**


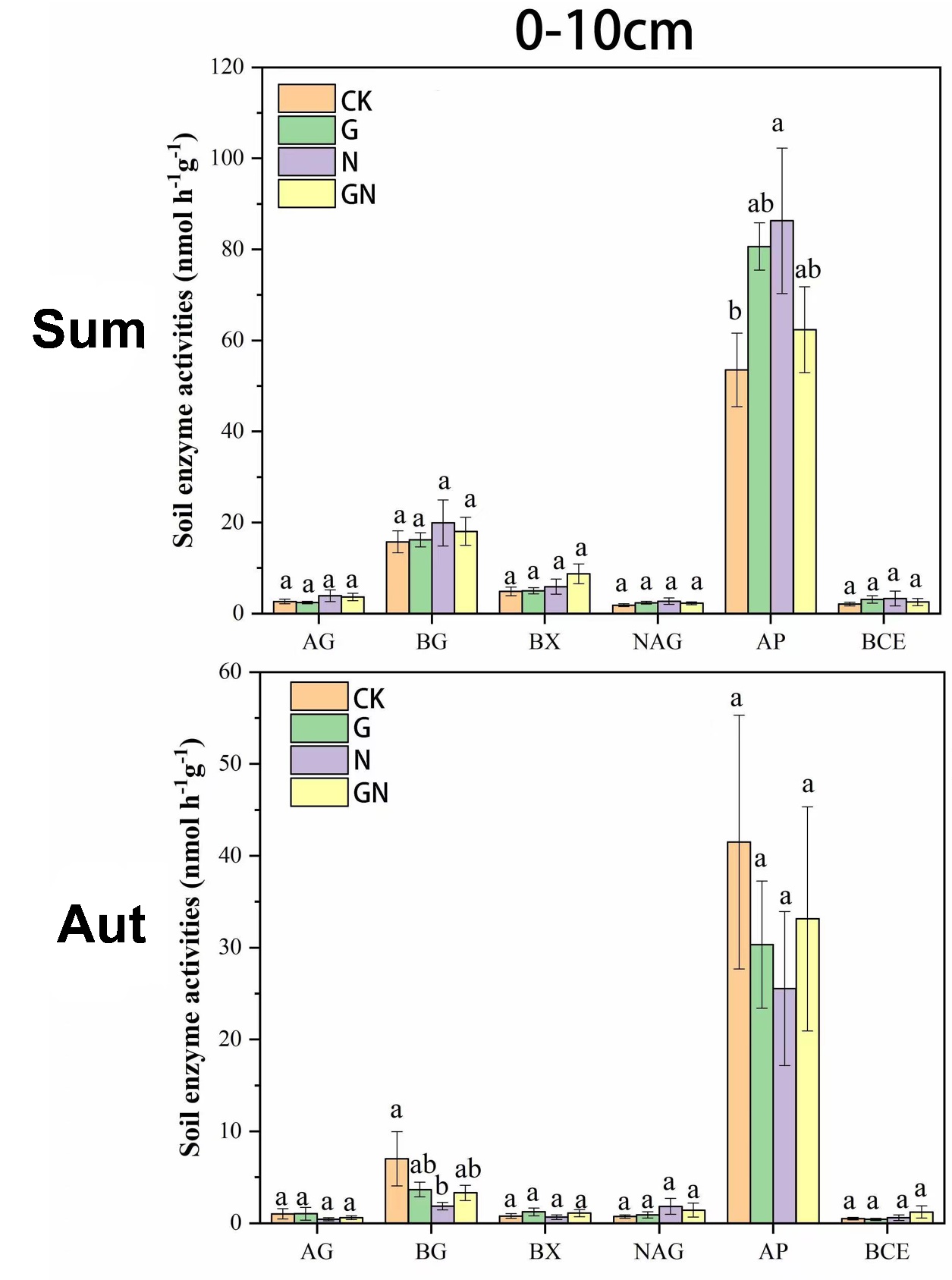


**Fig. S1** The activities of soil extracellular enzyme with each treatment group in summer and autumn. Bars show mean ± standard error and different lowercase letters indicate significant difference at p ≤ 0.05 using post-hoc pairwise contrasts. AG, α-glucosidase; BG, β-glucosidase; BX, β-Xylosidase; NAG, N-acetyl-β-glucosaminidase; ALP, alkaline phosphatase; BCE, β-D-cellobioside; CK: Control; G: Glyphosate addition; N: Chemical nitrogen addition; GN: Glyphosate and nitrogen addition; Sum: summer; Aut: autumn; Data that do not share a letter are significantly different between treatments (P < 0.05).


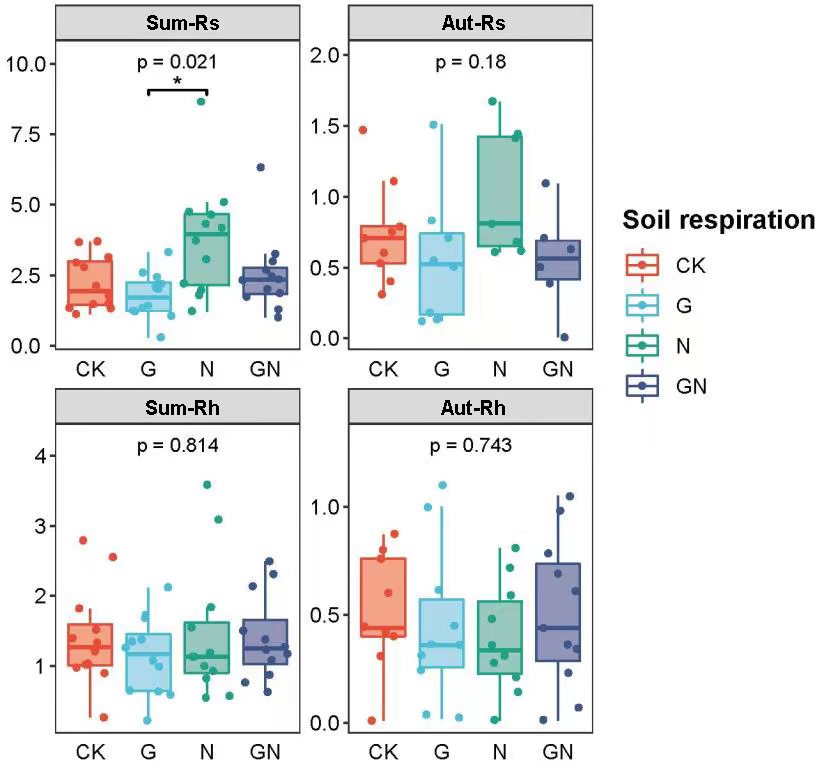


**Fig. S2** Soil respiration (R_S_) and soil heterotrophic respiration (R_H_) with each treatment group in summer and autumn. CK: Control; G: Glyphosate addition; N: Chemical nitrogen addition; GN: Glyphosate and nitrogen addition; Sum: summer; Aut: autumn.


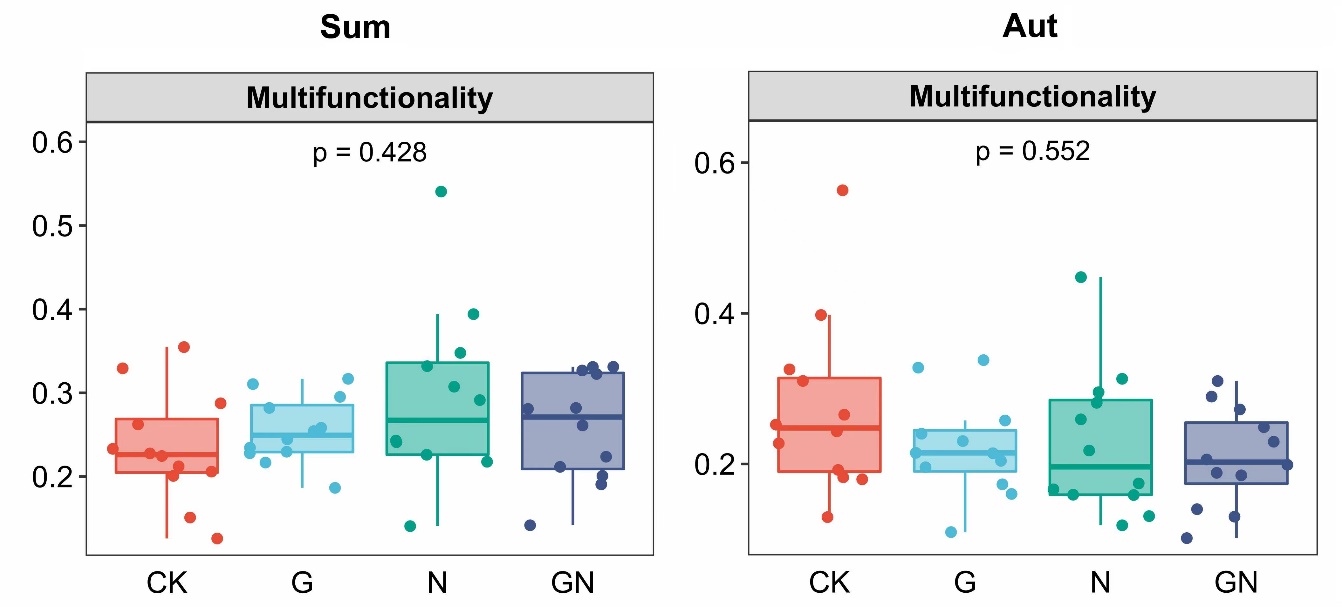


**Fig. S3** Soil multifunctionality with each treatment group. CK: Control; G: Glyphosate addition; N: Chemical nitrogen addition; GN: Glyphosate and nitrogen addition; Sum: summer; Aut: autumn.


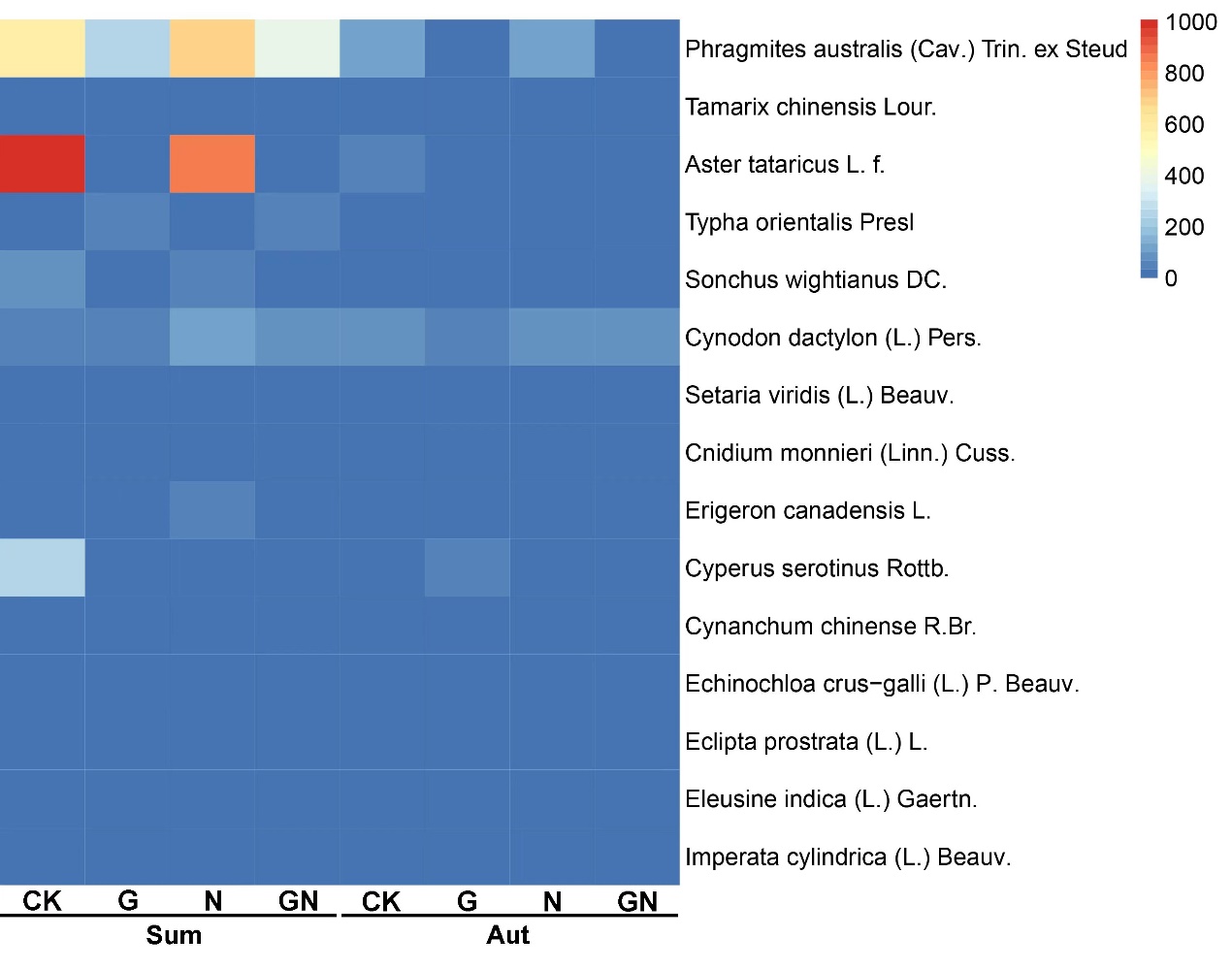


**Fig. S4** Plant community composition with each treatment group in summer and autumn. CK: Control; G: Glyphosate addition; N: Chemical nitrogen addition; GN: Glyphosate and nitrogen addition; Sum: summer; Aut: autumn.


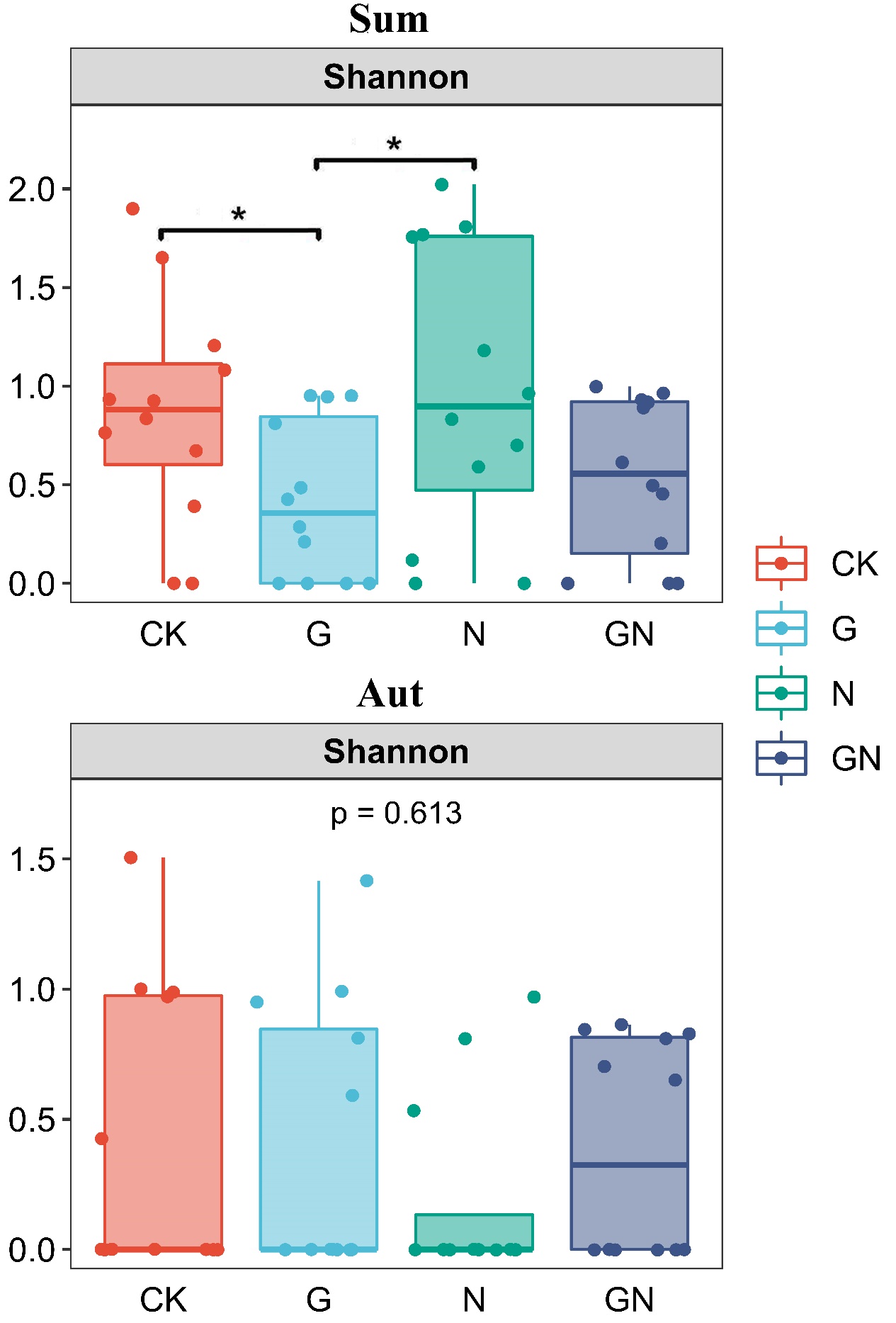


**Fig. S5** Shannon indices with each treatment group in summer and autumn. CK: Control; G: Glyphosate addition; N: Chemical nitrogen addition; GN: Glyphosate and nitrogen addition; Sum: summer; Aut: autumn.


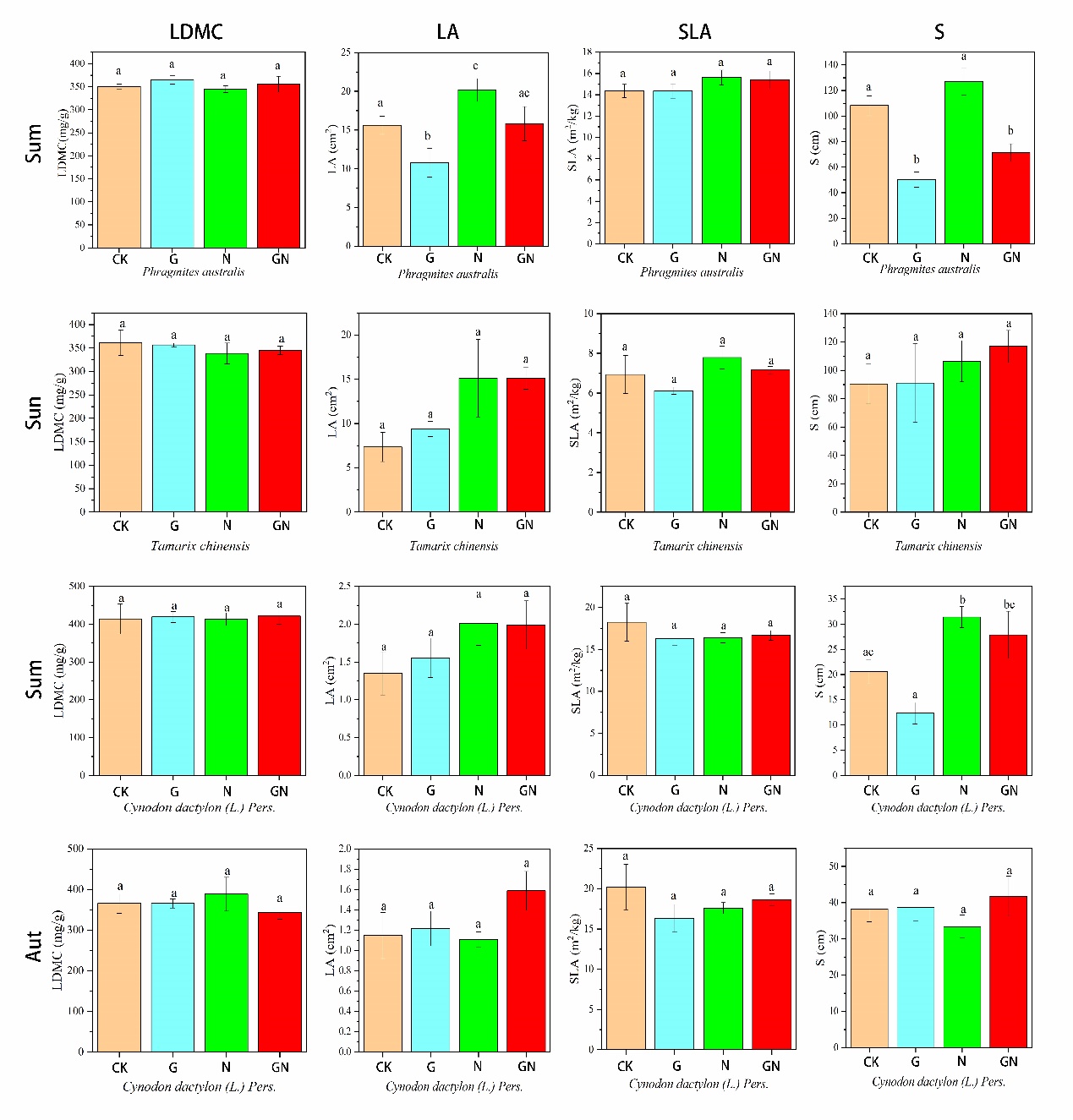


**Fig. S6** Plant functional traits with each treatment group in summer and autumn. CK: Control; G: Glyphosate addition; N: Chemical nitrogen addition; GN: Glyphosate and nitrogen addition; Sum: summer; Aut: autumn; LDMC: leaf dry matter content; LA: leaf area; SLA: specific leaf area; S: plant stature; Data that do not share a letter are significantly different between treatments (P < 0.05).


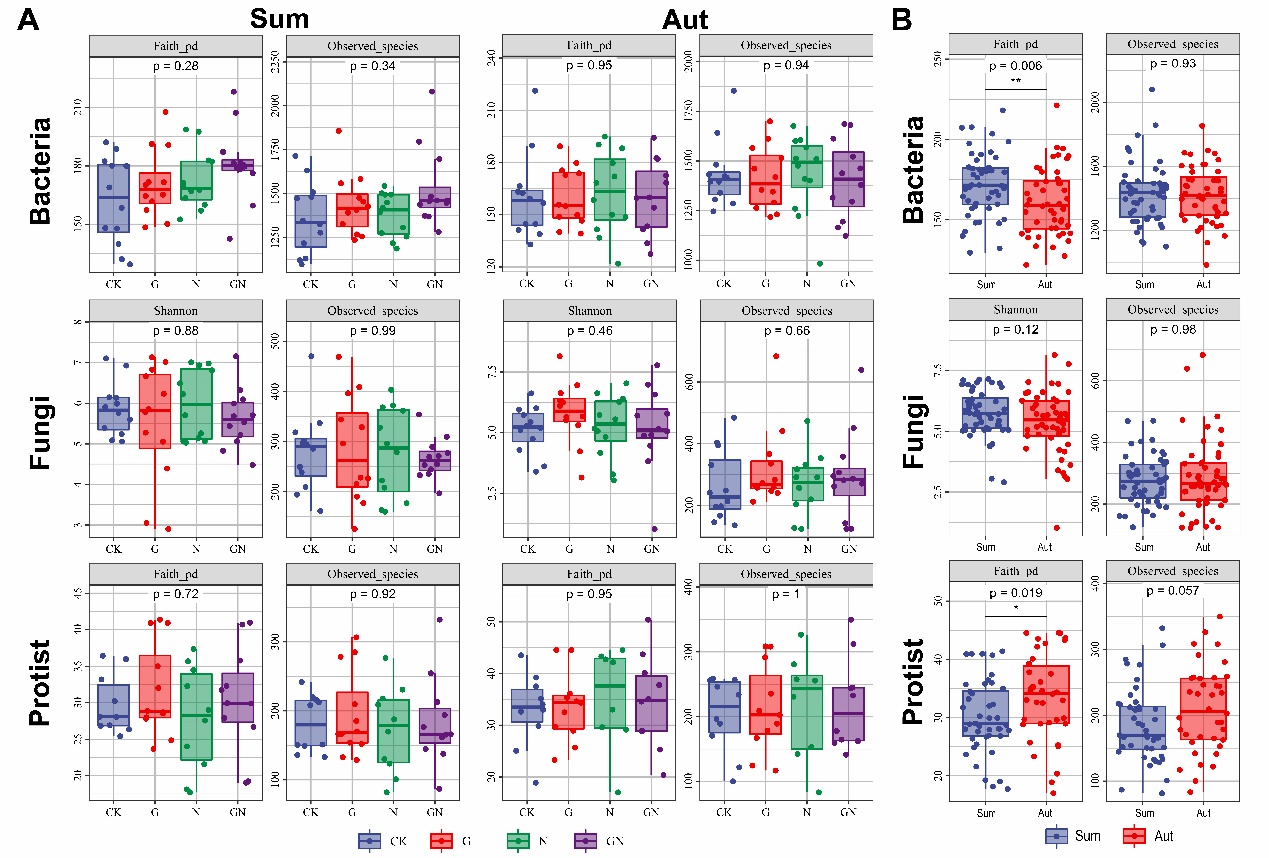


**Fig. S7** Alpha diversity indices of bacteria, fungi and protist with different experimental treatment and seasonal variation. CK: Control; G: Glyphosate addition; N: Chemical nitrogen addition; GN: Glyphosate and nitrogen addition; Sum: summer; Aut: autumn.


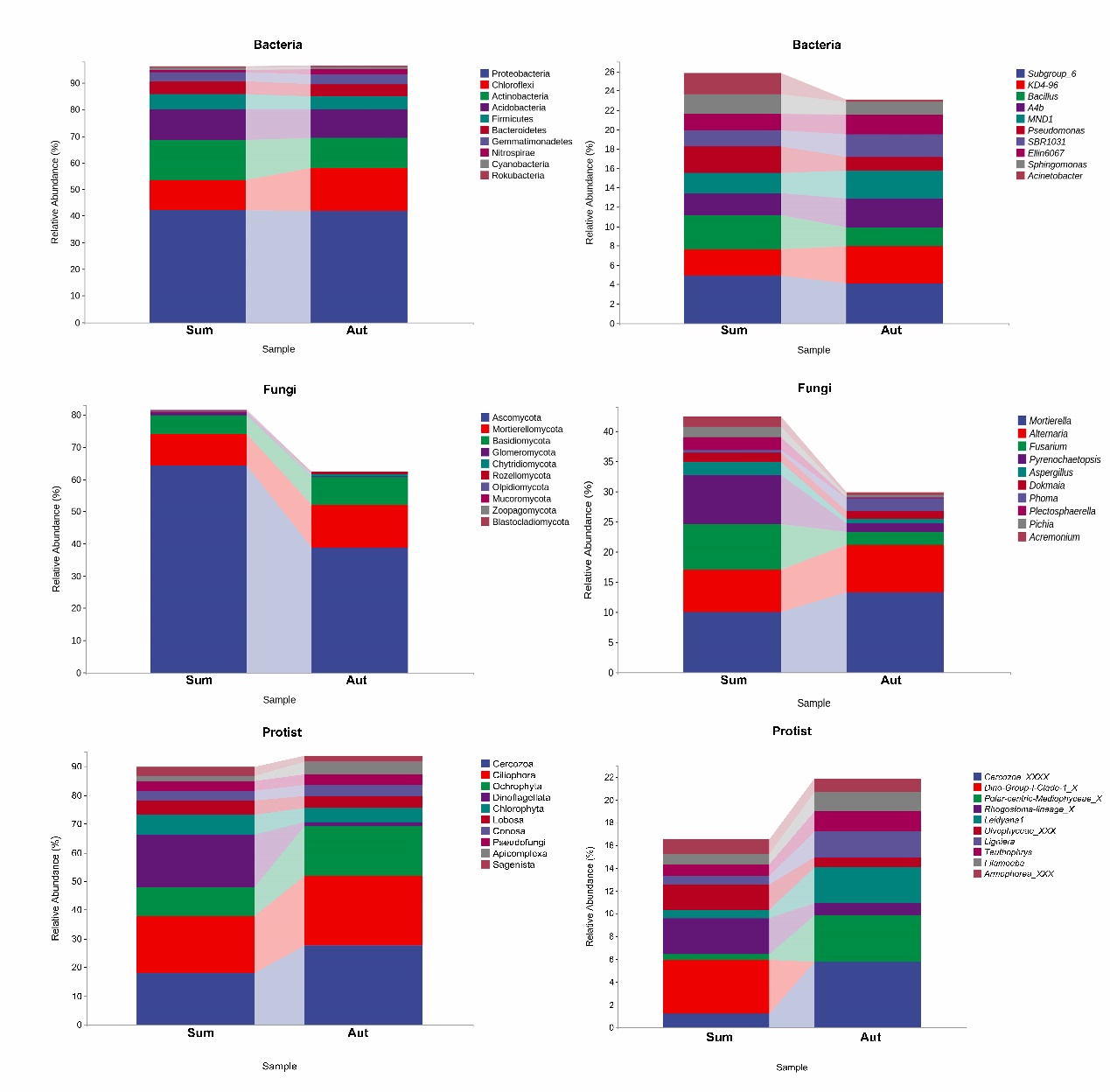


**Fig. S8** Composition of bacterial, fungal and protist communities in summer and autumn. Sum: summer; Aut: autumn.


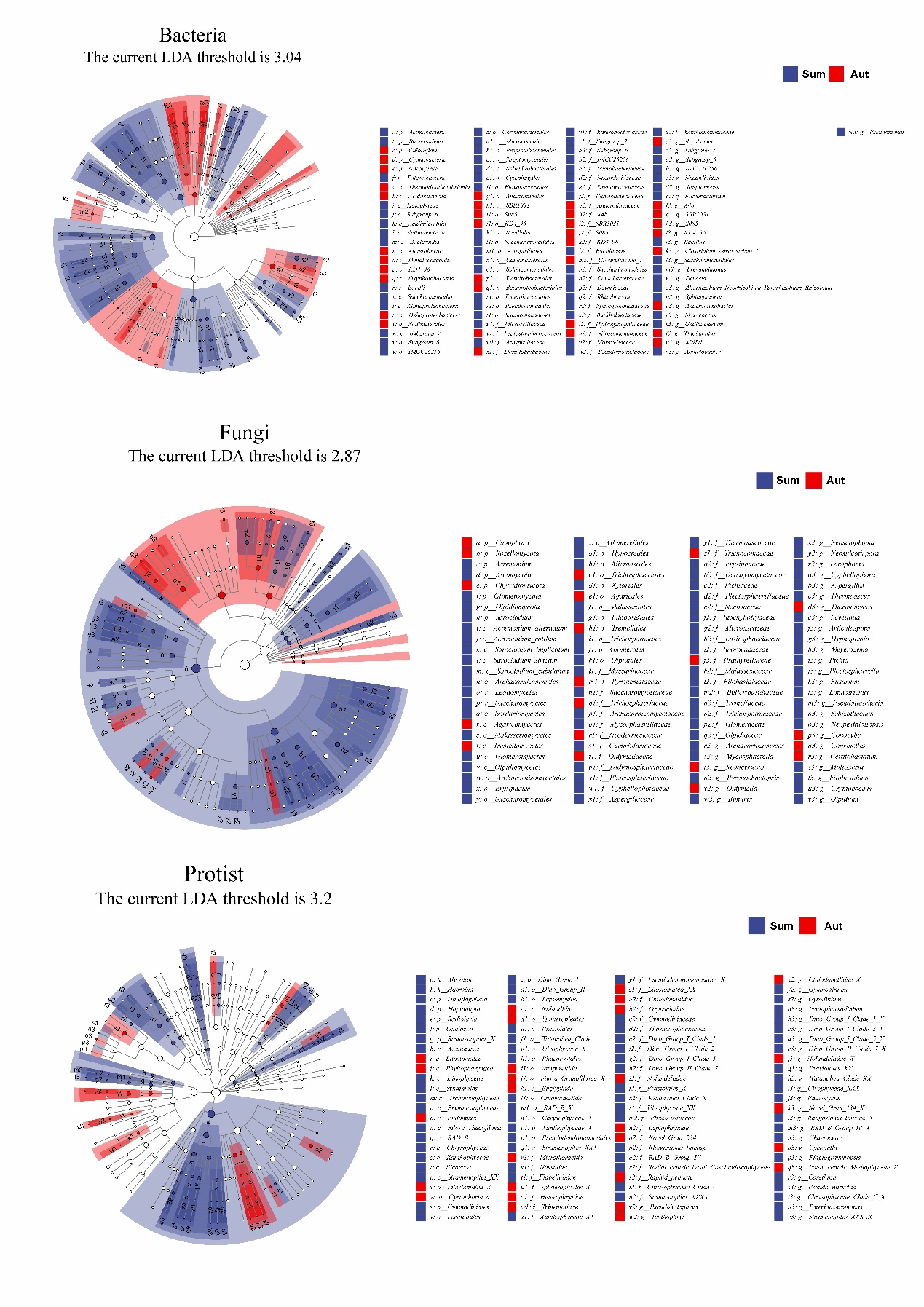


**Fig. S9** LEfSe analysis at multiple taxonomic levels comparing microbial community structure in summer and autumn. Cladogram illustrating the taxonomic groups explaining the most variation among communities. Each ring represents a taxonomic level, with phylum (p_), class (c_), order (o_) and family (f_) emanating from the center to the periphery. Sum: summer; Aut: autumn.


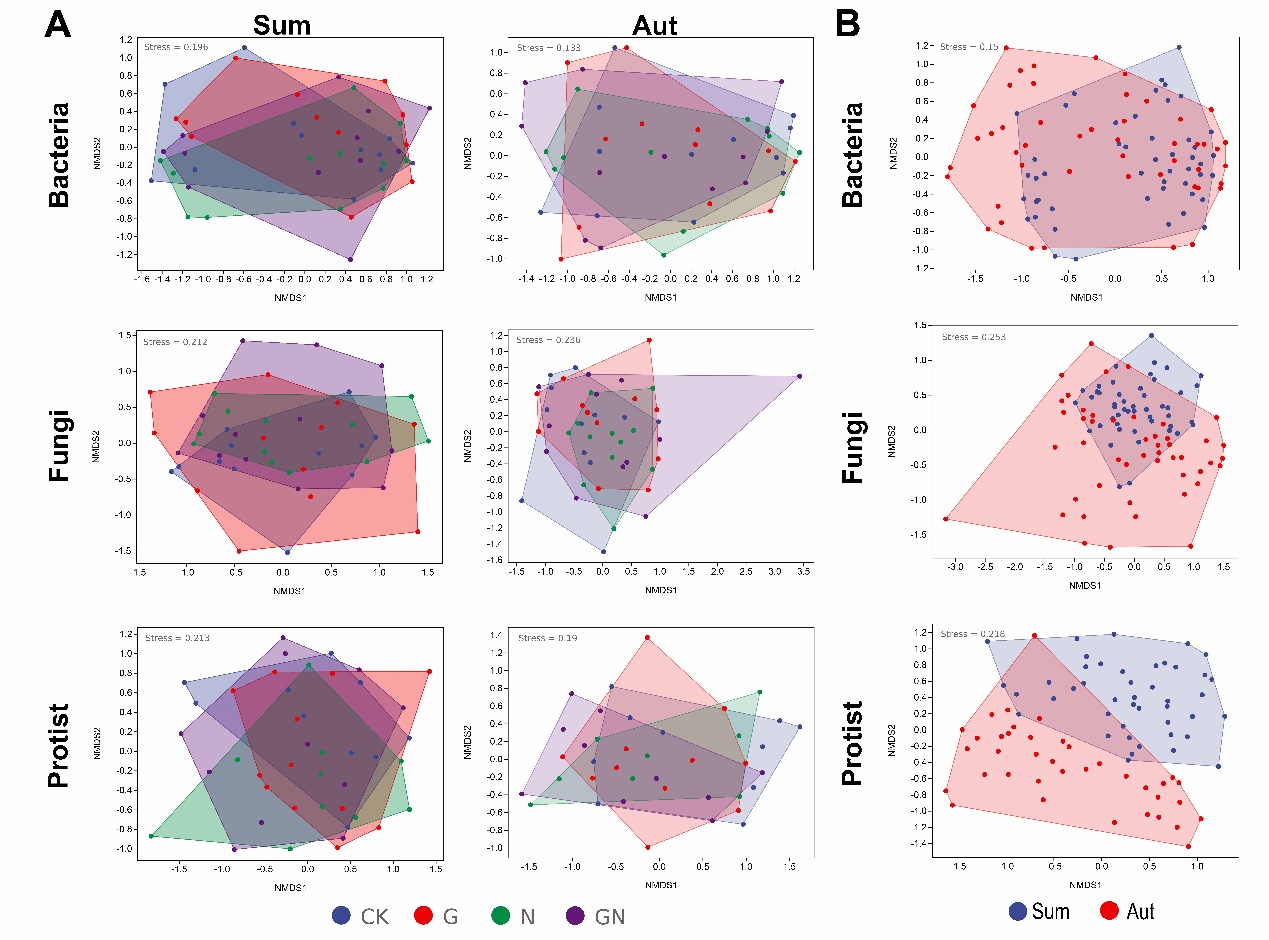


**Fig. S10** Taxonomic composition and the two-dimensional nonmetric multidimensional scaling (NMDS) ordinations show bacterial, fungal and protist communities between different experimental treatment in summer and autumn. CK: Control; G: Glyphosate addition; N: Chemical nitrogen addition; GN: Glyphosate and nitrogen addition; Sum: summer; Aut: autumn.


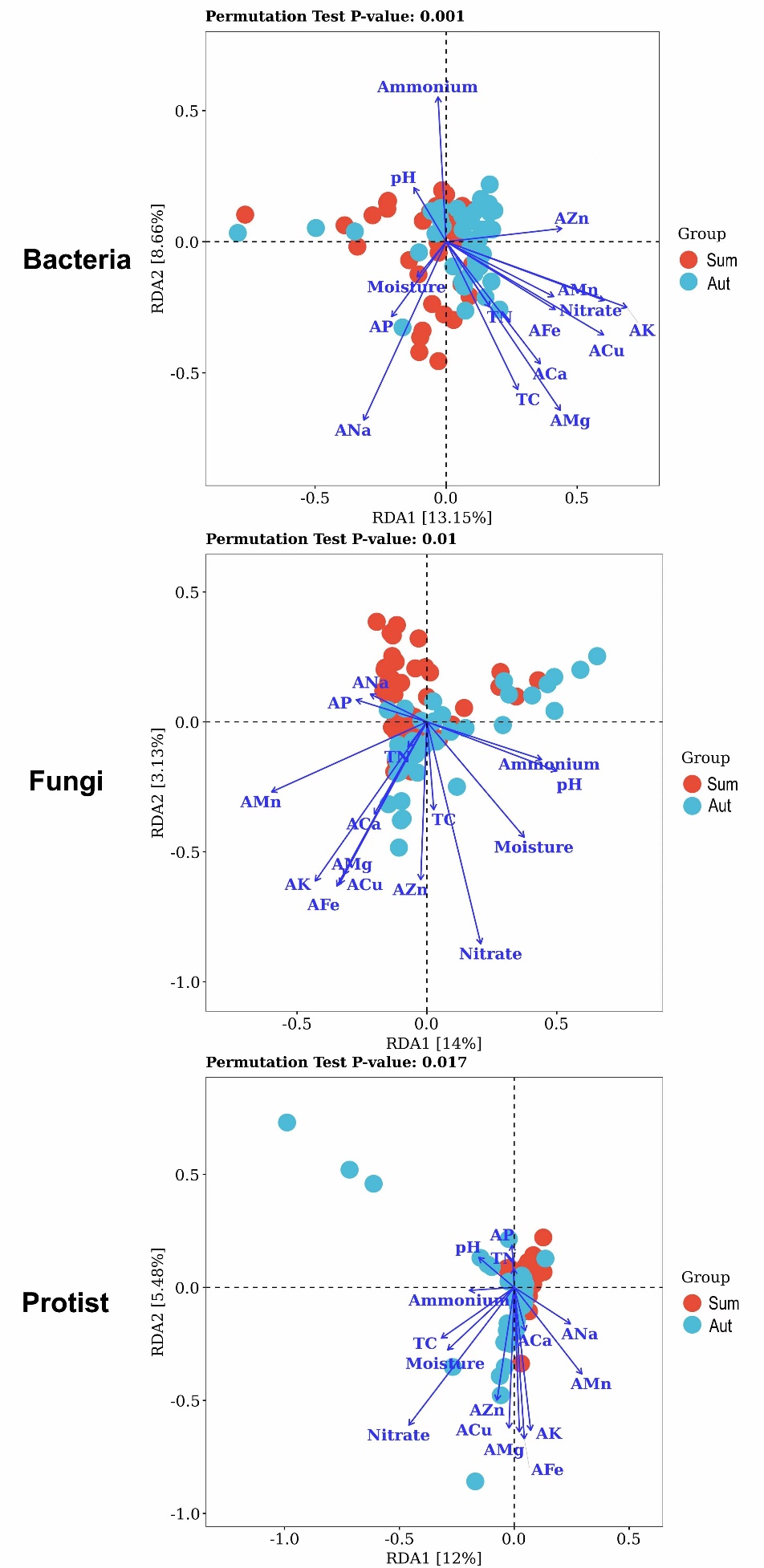


**Fig. S11** Redundancy analysis (RDA) compares the microbial community structure and environmental factors (arrows). Sum: summer; Aut: autumn.

Table S1. Ecosystem variables used to quantify regulating, supporting, and provisioning ecosystem services.

| Type | Ecosystem service | Ecosystem variable |
| --- | --- | --- |
| Regulating | Organic matter decomposition | Activity of α-glucosidase |
|  |  | Activity of β-glucosidase |
|  |  | Activity of β-Xylosidase |
|  |  | Activity of N-acetyl-β-glucosaminidase |
|  |  | Activity of alkaline phosphatase |
|  |  | Activity of β-D-cellobioside |
|  | Soil carbon cycling | Soil respiration |
|  |  | Soil heterotrophic respiration |
| Supporting | Soil fertility | Total nitrogen |
|  |  | Total carbon |
|  |  | Available potassium |
|  |  | Available calcium |
|  |  | Available magnesium |
|  |  | Available sodium |
|  |  | Available copper |
|  |  | Available iron |
|  |  | Available manganese |
|  |  | Available zinc |
|  |  | Available phosphorus |
|  |  | pH |

Table S2. Mean (±1 standard error) measures for soil variables with nitrogen fertilizer and glyphosate addition treatments in summer and autumn.

|  | Summer | | | | Autumn | | | |
| --- | --- | --- | --- | --- | --- | --- | --- | --- |
| Soil variables | CK | G | N | GN | CK | G | N | GN |
| Total nitrogen (%) | 0.04±0.00 a | 0.04±0.00 a | 0.03±0.00 a | 0.04±0.00 a | 0.04±0.00 a | 0.03±0.00 a | 0.03±0.00 a | 0.03±0.00 a |
| Total carbon (%) | 1.47±0.07 a | 1.47±0.06 a | 1.44±0.07 a | 1.44±0.08 a | 1.63±0.07 a | 1.52±0.06 ab | 1.48±0.07 ab | 1.42±0.06 b |
| Available potassium（mg/kg） | 69.42±6.92 a | 70.73±3.77 a | 59.08±4.69 a | 64.82±5.36 a | 83.89±11.46 a | 76.76±6.85 a | 76.78±11.18 a | 69.21±5.85 a |
| Available calcium（mg/kg） | 4040.10±144.28 a | 4114.18±117.99 a | 4110.24±127.78 a | 4146.27±124.81 a | 4129.03±139.49 a | 4082.95±89.36 a | 4057.39±88.09 a | 4140.72±76.17 a |
| Available magnesium（mg/kg） | 376.50±33.55 a | 371.98±27.25 a | 419.45±38.22 a | 382.23±36.28 a | 465.09±57.59 a | 409.05±36.19 a | 448.69±50.76 a | 392.67±31.68 a |
| Available sodium（mg/kg） | 176.15±26.61 b | 178.42±37.20 b | 291.66±46.55 a | 192.40±36.28 ab | 130.00±16.15 a | 124.46±19.97 a | 138.31±15.37 a | 119.05±12.51 a |
| Available copper（mg/kg） | 1.56±0.19 a | 1.54±0.11 a | 1.54±0.16 a | 1.57±0.22 a | 2.58±0.46 a | 1.94±0.23 a | 2.13±0.37 a | 1.78±0.26 a |
| Available iron（mg/kg） | 16.11±0.98 a | 15.77±0.77 a | 18.54±1.31 a | 16.61±2.06 a | 23.89±5.24 a | 22.84±5.88 a | 25.40±5.90 a | 19.95±4.88 a |
| Available manganese（mg/kg） | 9.57±0.98 a | 10.12±0.65 a | 10.15±0.75 a | 10.23±1.10 a | 9.66±1.06 a | 8.33±1.02 a | 8.92±0.93 a | 7.62±1.09 a |
| Available zinc（mg/kg） | 0.79±0.06 a | 0.84±0.06 a | 0.84±0.05 a | 0.82±0.07 a | 3.32±0.74 a | 2.76±0.45 a | 2.67±0.39 a | 2.79±0.47 a |
| Available phosphorus（mg/kg） | 9.56±1.06 a | 10.50±1.03 a | 10.66±2.39 a | 8.90±1.27 a | 8.47±1.07 ab | 9.88±1.54 a | 6.33±0.47 b | 7.15±0.62 ab |
| glyphosate (mg/kg) | 0.30±0.13 a | 0.66±0.49 a | 0.15±0.13 a | 0.54±0.26 a | 0.25±0.17 a | 0.30±0.17 a | 0.35±0.22 a | 0.21±0.12 a |
| Moisture (%) | 22.73±1.92 a | 25.06±1.77 a | 27.97±4.34 a | 23.27±2.46 a | 29.58±1.23 a | 31.41±2.18 a | 30.99±1.75 a | 33.40±2.10 a |
| pH | 8.25±0.03 a | 8.28±0.02 a | 8.34±0.04 a | 8.24±0.02 a | 8.46±0.03 a | 8.36±0.02 b | 8.36±0.03 b | 8.41±0.03 ab |
| NO_3_^-^ (mg/kg) | 0.23±0.05 a | 0.25±0.07 a | 0.20±0.05 a | 0.34±0.06 a | 0.82±0.03 ab | 0.81±0.04 b | 0.88±0.03 ab | 0.93±0.06 a |
| NH_4_^+^ (mg/kg) | 1.99±0.76 a | 3.31±0.90 a | 3.45±0.94 a | 3.63±1.04 a | 4.43±0.13 a | 4.45±0.13 a | 4.57±0.16 a | 4.53±0.19 a |
| Soil temperature | 26.21±0.41a | 26.18±0.51 a | 26.03±0.942a | 25.97±0.43 a | 19.48±0.61a | 19.44±0.61a | 19.84±0.74a | 20.52±0.91a |

Table S3. Mean (±1 standard error) measures for soil variables in summer and autumn.

| Soil variables | Summer | Autumn |
| --- | --- | --- |
| Total nitrogen (%) | 0.04±0.00 a | 0.03±0.00 a |
| Total carbon (%) | 1.45±0.03 a | 1.51±0.03 a |
| Available potassium（mg/kg） | 66.01±2.65 b | 76.66±4.51 a |
| Available calcium（mg/kg） | 4102.70±62.70 a | 4102.52±49.23 a |
| Available magnesium（mg/kg） | 387.54±16.71 a | 428.88±22.32 a |
| Available sodium（mg/kg） | 209.66±19.36 a | 127.96±7.92 b |
| Available copper（mg/kg） | 1.55±0.08 b | 2.11±0.17 a |
| Available iron（mg/kg） | 16.76±0.68 b | 23.02±2.67 a |
| Available manganese（mg/kg） | 10.02±0.43 a | 8.63±0.51 b |
| Available zinc（mg/kg） | 0.82±0.03 b | 2.88±0.26 a |
| Available phosphorus（mg/kg） | 9.90±0.75 a | 7.96±0.53 b |
| glyphosate (mg/kg) | 0.41±0.14 a | 0.28±0.08 a |
| Moisture (%) | 24.76±1.39 b | 31.34±0.92 a |
| pH | 8.28±0.02 b | 8.40±0.02 a |
| NO_3_^-^ (mg/kg) | 0.25±0.03 b | 0.86±0.02 a |
| NH_4_^+^ (mg/kg) | 3.15±0.48 b | 4.50±0.07 a |
| Soil temperature | 26.09±1.49 a | 19.82±2.49 b |

Table S4 The relative importance of experimental treatment (nitrogen and glyphosate addition) and seasonal variation for the total bacterial, fungal and protist community structure in the samples investigated in this study as revealed by PERMANOVA.

|  |  | Bacteria | | Fungi | | Protist | |
| --- | --- | --- | --- | --- | --- | --- | --- |
| Parameter | Df | R² | P | R² | P | R² | P |
| Experimental treatments | 3 | 0.028 | 0.727 | 0.029 | 0.559 | 0.036 | 0.382 |
| Seasonal variation | 1 | 0.039 | 0.001** | 0.044 | 0.001** | 0.047 | 0.001** |
| Experimental treatments×seasonal variation | 3 | 0.024 | 0.980 | 0.027 | 0.849 | 0.032 | 0.881 |

Table S5 Mantel test was used to analyze the correlations between soil microbial community and physicochemical properties.

|  | Bacteria | | Fungi | | Protist | |
| --- | --- | --- | --- | --- | --- | --- |
|  | R | P | R | P | R | P |
| Total nitrogen | 0.061 | 0.034 | 0.045 | 0.169 | 0.023 | 0.203 |
| Total carbon | 0.160 | 0.001 | 0.022 | 0.308 | 0.089 | 0.002 |
| Available potassium | 0.150 | 0.001 | 0.148 | 0.006 | 0.087 | 0.006 |
| Available calcium | 0.133 | 0.001 | 0.034 | 0.238 | 0.030 | 0.183 |
| Available magnesium | 0.320 | 0.001 | 0.146 | 0.002 | 0.152 | 0.001 |
| Available sodium | 0.135 | 0.004 | -0.095 | 0.961 | 0.070 | 0.029 |
| Available copper | 0.217 | 0.001 | 0.257 | 0.001 | 0.112 | 0.003 |
| Available iron | 0.184 | 0.001 | 0.297 | 0.001 | 0.096 | 0.011 |
| Available manganese | 0.114 | 0.003 | 0.131 | 0.010 | 0.099 | 0.003 |
| Available zinc | 0.080 | 0.027 | 0.222 | 0.001 | 0.131 | 0.003 |
| Available phosphorus | -0.003 | 0.494 | -0.023 | 0.641 | -0.033 | 0.820 |
| Moisture | 0.024 | 0.284 | -0.060 | 0.835 | 0.066 | 0.039 |
| pH | 0.022 | 0.252 | 0.006 | 0.450 | 0.069 | 0.017 |
| NO_3_^-^ | 0.182 | 0.001 | 0.138 | 0.001 | 0.268 | 0.001 |
| NH_4_^+^ | 0.046 | 0.082 | -0.068 | 0.942 | 0.034 | 0.125 |

**Table S6** Topological properties of co-occurrence networks.

|  | **Summer** | **Autumn** |
| --- | --- | --- |
| **Number of nodes** | 352 | 398 |
| **Number of edges** | 1125 | 1718 |
| **Edges/nodes** | 3.19 | 4.32 |
| **Avg number of neighbors** | 6.745 | 8.953 |
| **Network diameter** | 11 | 10 |
| **Network radius** | 6 | 6 |
| **characteristic path length** | 3.944 | 3.527 |
| **clustering coefficient** | 0.265 | 0.268 |
| **Network density** | 0.021 | 0.023 |
| **Network heterogeneity** | 1.433 | 1.277 |
| **Network centralization** | 0.261 | 0.158 |
| **connected components** | 11 | 9 |
| **Positive correlation** | 50.68％ | 33.93％ |
| **Negative correlation** | 49.32％ | 66.07％ |

**Table S7** Kinless hubs, Connector and Provincial hubs distribution in co-occurrence network of summer and autumn.

|  | type | Kingdom | Phylum | Class | Order | Family | Genus |
| --- | --- | --- | --- | --- | --- | --- | --- |
| **Sum** | Kinless hubs | Fungi | unclassified_Fungi | unclassified_Fungi | unclassified_Fungi | unclassified_Fungi | unclassified_Fungi |
|  | Provincial hubs | Fungi | Ascomycota | Eurotiomycetes | Eurotiales | Aspergillaceae | Aspergillus |
|  | Provincial hubs | Bacteria | Acidobacteria | Blastocatellia_(Subgroup_4) | Pyrinomonadales | Pyrinomonadaceae | g__RB41 |
|  | Provincial hubs | Bacteria | Latescibacteria | Latescibacteria | Latescibacteria | Latescibacteria | Latescibacteria |
|  | Provincial hubs | Bacteria | Proteobacteria | Alphaproteobacteria | Dongiales | Dongiaceae | Dongia |
|  | Provincial hubs | Bacteria | Proteobacteria | Alphaproteobacteria | Sphingomonadales | Sphingomonadaceae | Sphingomonas |
|  | Provincial hubs | Bacteria | Proteobacteria | Alphaproteobacteria | Rhizobiales | Methyloligellaceae | uncultured |
|  | Provincial hubs | Bacteria | Chloroflexi | KD4-96 | KD4-96 | KD4-96 | KD4-96 |
|  | Provincial hubs | Bacteria | Acidobacteria | Subgroup_6 | Subgroup_6 | Subgroup_6 | Subgroup_6 |
|  | Provincial hubs | Bacteria | Actinobacteria | MB-A2-108 | MB-A2-108 | MB-A2-108 | MB-A2-108 |
|  | Provincial hubs | Bacteria | Gemmatimonadetes | Gemmatimonadetes | Gemmatimonadales | Gemmatimonadaceae | uncultured |
| **Aut** | Provincial hubs | Bacteria | Proteobacteria | Alphaproteobacteria | Rhizobiales | Methyloligellaceae | uncultured |
|  | Provincial hubs | Bacteria | Proteobacteria | Alphaproteobacteria | Rhizobiales | Methyloligellaceae | Methyloceanibacter |
|  | Provincial hubs | Bacteria | Gemmatimonadetes | Gemmatimonadetes | Gemmatimonadales | Gemmatimonadaceae | uncultured |
|  | Provincial hubs | Bacteria | Proteobacteria | Gammaproteobacteria | Betaproteobacteriales | Nitrosomonadaceae | Ellin6067 |
|  | Connector | Bacteria | Actinobacteria | Thermoleophilia | uncultured | uncultured | uncultured |
|  | Connector | Bacteria | Proteobacteria | Gammaproteobacteria | Betaproteobacteriales | Burkholderiaceae | uncultured |
|  | Connector | Bacteria | Actinobacteria | MB-A2-108 | MB-A2-108 | MB-A2-108 | MB-A2-108 |
|  | Connector | Bacteria | Chloroflexi | KD4-96 | KD4-96 | KD4-96 | KD4-96 |

Table S8 The relative contribution (%) of individual mechanism of soil microbial community assembly in different experimental treatment and seasonal variation.

|  | Summer | | | | Autumn | | | |  |  |
| --- | --- | --- | --- | --- | --- | --- | --- | --- | --- | --- |
| **Bacteria** | CK | G | N | GN | CK | G | N | GN | Sum | Aut |
| Variable selection (βΝΤΙ> +2) | 0 | 1.52 | 4.55 | 12.12 | 4.55 | 4.55 | 31.82 | 31.82 | 4.79 | 18.09 |
| Homogenous selection (βΝΤΙ< -2) | 45.45 | 48.48 | 54.55 | 39.39 | 31.82 | 36.36 | 33.33 | 34.85 | 48.65 | 34.57 |
| **Total selection (deterministic processes)** | **45.45** | **50** | **59.1** | **51.51** | **36.37** | **40.91** | **65.15** | **66.67** | **53.45** | **52.66** |
| Dispersal limitation  (βΝΤΙ<\|2\| & RC> +0.95) | 45.45 | 42.42 | 36.36 | 40.91 | 54.55 | 56.06 | 31.82 | 25.75 | 40.96 | 39.27 |
| Dispersal homogenizing  (βΝΤΙ<\|2\| & RC< -0.95) | 0.00 | 0 | 1.52 | 0 | 0 | 0 | 0 | 0 | 0.09 | 0.45 |
| Undominated  (βΝΤΙ<\|2\| & RC< \|0.95\|) | 9.10 | 7.58 | 3.02 | 7.58 | 9.08 | 3.03 | 3.03 | 7.58 | 5.5 | 7.62 |
| **Total neutral (stochastic processes)** | **55.55** | **50** | **40.9** | **48.49** | **63.63** | **59.09** | **34.85** | **33.33** | **46.55** | **47.34** |
| **fungi** | CK | G | N | GN | CK | G | N | GN | Jun | Nov |
| Variable selection (βΝΤΙ> +2) | 0 | 0 | 0 | 0 | 9.10 | 1.52 | 0 | 7.58 | 0 | 4.34 |
| Homogenous selection (βΝΤΙ< -2) | 77.27 | 59.09 | 84.85 | 100 | 42.42 | 21.20 | 45.45 | 12.12 | 80.23 | 30.23 |
| **Total selection (deterministic processes)** | **77.27** | **59.09** | **84.85** | **100** | **51.52** | **22.72** | **45.45** | **19.7** | **80.23** | **34.57** |
| Dispersal limitation  (βΝΤΙ<\|2\| & RC> +0.95) | 9.09 | 30.3 | 4.55 | 0 | 27.27 | 60.61 | 31.82 | 68.18 | 10.37 | 47.7 |
| Dispersal homogenizing  (βΝΤΙ<\|2\| & RC< -0.95) | 0 | 0 | 0 | 0 | 0 | 0 | 0 | 0 | 0 | 0 |
| Undominated  (βΝΤΙ<\|2\| & RC< \|0.95\|) | 13.64 | 10.61 | 10.60 | 0 | 21.21 | 16.67 | 22.73 | 12.12 | 9.4 | 17.73 |
| **Total neutral (stochastic processes)** | **22.73** | **40.91** | **15.15** | **0** | **48.48** | **77.28** | **54.55** | **80.3** | **19.77** | **65.43** |
| **protist** | CK | G | N | GN | CK | G | N | GN | Jun | Nov |
| Variable selection (βΝΤΙ> +2) | 0 | 7.58 | 2.22 | 0 | 13.33 | 0 | 0 | 8.89 | 1.48 | 5.53 |
| Homogenous selection (βΝΤΙ< -2) | 24.45 | 45.45 | 20 | 9.09 | 2.22 | 25.45 | 17.86 | 22.22 | 23.47 | 17.27 |
| **Total selection (deterministic processes)** | **24.45** | **53.03** | **22.22** | **9.09** | **15.55** | **25.45** | **17.86** | **31.11** | **24.95** | **22.8** |
| Dispersal limitation  (βΝΤΙ<\|2\| & RC> +0.95) | 33.33 | 13.64 | 17.78 | 43.94 | 48.90 | 49.09 | 60.71 | 57.78 | 28.01 | 53.31 |
| Dispersal homogenizing  (βΝΤΙ<\|2\| & RC< -0.95) | 0 | 0 | 0 | 0 | 2.22 | 0 | 0 | 0 | 0 | 0.4 |
| Undominated  (βΝΤΙ<\|2\| & RC< \|0.95\|) | 42.22 | 33.33 | 60 | 46.97 | 33.33 | 25.46 | 21.43 | 11.11 | 47.04 | 23.49 |
| **Total neutral (stochastic processes)** | **75.55** | **46.97** | **77.78** | **90.91** | **84.45** | **74.55** | **74.54** | **68.89** | **75.05** | **77.2** |

**Experimental procedure**

**Statistical analysis**

The abundance-weighted beta nearest taxon index (βNTI), which is the difference in a standard phylogenetic deviation between the observed mean nearest taxon distance (MNTD) and the mean of the random null expectation (999 for the number of randomizations), was also calculated using the R package picante's "comdistnt" function (Stegen et al., 2012). Following that, the relative contribution of the assembly process and underlying processes was assessed using the βNTI and modified Raup-Crick (RC_Bray_) metrics (Stegen et al., 2015). When |βNTI| exceeds 2, the community is predominantly built by deterministic processes, such as homogeneous selection (βNTI < -2) and heterogeneous selection (NTI > 2). When |βNTI| is less than 2, the community is primarily driven by stochastic processes, specifically, dispersal (including homogenizing dispersal [RC_Bray_ < -0.95] and dispersal limitation [RC_Bray_ > +0.95]) and undominated processes [|RC_Bray_| < 0.95], which include the weak influence of selection, dispersal, diversification, and drift (Chase et al., 2011).

**References**

Chase, J. M., Kraft, N. J. B., Smith, K. G., Vellend, M., and Inouye, B. D., (2011). Using null models to disentangle variation in community dissimilarity from variation in α‐diversity. *Ecosphere.* 2, 1-11.

Stegen, J. C., Lin, X., Konopka, A. E., and Fredrickson, J. K., (2012). Stochastic and deterministic assembly processes in subsurface microbial communities. *ISME J.* 6, 1653-1664.

Stegen, J. C., Lin, X., Fredrickson, J. K., and Konopka, A. E., (2015). Estimating and mapping ecological processes influencing microbial community assembly. *Front. Microbiol*. 6, 370.
